# Chinese herbal medicine combined with oxaliplatin-based chemotherapy for advanced gastric cancer: A systematic review and meta-analysis of contributions of specific medicinal materials to tumor response

**DOI:** 10.3389/fphar.2022.977708

**Published:** 2022-08-25

**Authors:** Ying Tan, Heping Wang, Bowen Xu, Xiaoxiao Zhang, Guanghui Zhu, Yuansha Ge, Taicheng Lu, Ruike Gao, Jie Li

**Affiliations:** ^1^ Department of Oncology, Guang’anmen Hospital, China Academy of Chinese Medical Sciences, Beijing, China; ^2^ Graduate School, Beijing University of Chinese Medicine, Beijing, China

**Keywords:** advanced gastric cancer, Chinese herbal medicine, oxaliplatin, meta-analysis, efficacy, tumor response, synergistic action

## Abstract

**Introduction:** The incidence and mortality of gastric cancer ranks among the highest, and the 5-year survival rate of advanced gastric cancer (AGC) is less than 10%. Currently, chemotherapy is the main treatment for AGC, and oxaliplatin is an important part of the commonly used chemotherapy regimen for AGC. A large number of RCTs have shown that Chinese herbal medicine (CHM) combined with oxaliplatin-based chemotherapy can improve objective response rate (ORR) and disease control rate (DCR), reduce the toxic and side effects of chemotherapy. There is currently a lack of systematic evaluation of the evidence to account for the efficacy and safety of CHM combined with oxaliplatin-based chemotherapy in AGC. Therefore, we carried out this study and conducted the sensitivity analysis on the herbal composition to explore the potential anti-tumor efficacy.

**Methods:** Databases of PubMed, EMBASE, CENTRAL, Web of Science, the Chinese Biomedical Literature Database, the China National Knowledge Infrastructure, the Wanfang database, and the Chinese Scientific Journals Database were searched from their inception to April 2022. RCTs evaluating the efficacy of CHM combined with oxaliplatin-based chemotherapy on AGC were included. Stata 16 was used for data synthesis, RoB 2 for quality evaluation of included RCTs, and GRADE for quality of synthesized evidence. Additional sensitivity analysis was performed to explore the potential anti-tumor effects of single herbs and combination of herbs.

**Results:** Forty trials involving 3,029 participants were included. Most included RCTs were assessed as “Some concerns” of risk of bias. Meta-analyses showed that compare to oxaliplatin-based chemotherapy alone, that CHM combined with oxaliplatin-based chemotherapy could increase the objective response rate (ORR) by 35% [risk ratio (RR) = 1.35, 95% confidence intervals (CI) (1.25, 1.45)], and disease control rate (DCR) by 12% [RR = 1.12, 95% CI (1.08, 1.16)]. Subgroup analysis showed that compare to SOX, FOLFOX, and XELOX regimens alone, CHM plus SOX, CHM plus FOLFOX, and CHM plus XELOX could significantly increase the ORR and DCR. Sensitivity analysis identified seven herbs of Astragalus, Liquorice, Poria, Largehead Atractylodes, Chinese Angelica, Codonopsis, and Tangerine Peel with potentials to improve tumor response of oxaliplatin-based chemotherapy in AGC.

**Conclusion:** Synthesized evidence showed moderate certainty that CHM plus oxaliplatin-based chemotherapy may promote improvement in tumor response in AGC. CHM treatment is safe for AGC. Due to the poor quality of included RCTs and small samplesizes, the quality of synthesized evidence was not high. Specific combinations of herbs appeared to produce higher contributions to ORR than the herb individually. Each of this seven above mentioned herbs has been shown in experimental studies to potentially contribute to the improvement of tumor response. To support this conclusion, these seven herbs are worthy of further clinical research.

**Systematic Review Registration**: [http://www.crd.york.ac.uk/PROSPERO/display_record.php?RecordID=262595], identifier [CRD42022262595].

## 1 Introduction

According to Global Cancer Statistics 2020, there were 1,089,000 new cases and 769,000 mortality cases of gastric cancer (GC) globally, ranked second of incidence and mortality rate of all malignant tumors of digestive system (Sung et al., 2021). The number of GC cases in China accounts for 43.9% of the global total. About 50% of GC patients were in advanced stage at the initial diagnosis. Advanced gastric cancer (AGC) has a poor prognosis, with a median overall survival (OS) of 10–12 months (Digklia et al., 2016), and the 5-year survival rate is no more than 10% (Song et al., 2017). AGC generally have distant metastasis and local infiltration, and 50% of recurrent patients were local lymph node positive (Peng et al., 2010). It is reported that the OS of patients with metastatic GC after palliative chemotherapy is only 7–15 months, and the 5-year survival rate is only 2% (Leong, 2005; Ajani et al., 2007). In recent years, the incidence and death of GC are on the rise. Therefore, AGC has become one of the main diseases endangering human life and health (Johnston et al., 2019).

Chemotherapy is the standard first-line treatment for AGC patients, and palliative chemotherapy has a statistically significant advantage over best supportive care in improving survival in AGC patients (Wagner et al., 2006). The NCCN guidelines recommend that combined chemotherapy containing platinum and fluorouracil is preferred for AGC patients (Ajani et al., 2022). Oxaliplatin is a third-generation platinum-type anticancer drug. Clinical studies have proved that it has a significant inhibitory effect on locally AGC or AGC, and its efficacy is no less than that of cisplatin (Ajani et al., 2016; Smyth et al., 2016; Yoshino et al., 2018). The median progression-free survical (mPFS) of first-line chemotherapy is 4–6 months and median overall survival (mOS) is 10–15 months (Van Cutsem et al., 2006; Cunningham et al., 2008; Koizumi et al., 2008; Kang et al., 2009). Moreover, oxaliplatin is better tolerated than cisplatin and has a better synergistic effect with fluorouracil (Al-Batran et al., 2008). Based on the above advantages, oxaliplatin has become the main platinum drug in AGC chemotherapy, which is used to form SOX, XELOX and FOLFOX regimens. However, peripheral neurotoxicity is the main side effect of oxaliplatin, which incidence of peripheral neurotoxicity is higher than that of cisplatin (63% vs. 22%), especially grade 3 or 4 neuropathy (Al-Batran et al., 2008; Cunningham et al., 2008). The targeted drugs approved for the treatment of AGC are mainly anti-angiogenic drugs, including trastuzumab and ramucirumab. The mOS of trastuzumab in the treatment of AGC patients with positive HER-2 is 7.9 months, but the incidence of anemia and visceral bleeding, two kinds of serious adverse events (AEs), is about 19% (Thuss-Patience et al., 2017). In addition, HER-2 overexpression only accounts for 15%–20% of AGC (Joshi et al., 2021), and the benefit in OS of other drugs is unclear, and the selection of targeted drugs is limited. There is no evidence supporting survival benefit of immunotherapy alone in patients with AGC (Shitara et al., 2018; Bang et al., 2019; Van Cutsem et al., 2021). Therefore, despite the variety of treatment options for AGC, chemotherapy is still the best choice for AGC, and chemotherapy drugs are constantly updated and iterated. However, obstacles such as serious AEs of chemotherapy, poor quality of life (QoL) and short survival of patients with AGC have not been well solved, and the treatment of AGC is still a major challenge.

CHM has been widely used in east Asia to fight against tumors for a long time. In particular, CHM combined with chemotherapy has advantages of synergistic efficacy and toxicity reduction, improving QoL and enhancing immune function (Cheng et al., 2021). A meta-analysis of 2,670 patients with AGC found that patients with astragalus-containing Chinese medicine combined with platinum-containing chemotherapy had better objective response rate (ORR) [risk ratio (RR) = 1.24, 95% confidence interval (CI): 1.15–1.34) ] and disease control rate (DCR) (RR = 1.10, 95% CI: 1.06–1.14), the AEs caused by chemotherapy were significantly reduced, and the QoL was significantly improved (Cheng et al., 2021). There was no previous meta-analysis of oxaliplatin-based chemotherapy regimen plus CHM, but some high quality clinical studies have demonstrated the important role of CHM combined with oxaliplatin. Yiqi Huoxue Jiedu formula combined with XELOX regimen could improve DCR (60.78% vs. 41.67%) (Hu, 2011). Shenlian capsules combined with SOX chemotherapy intervention in 157 patients with AGC, the ORR and DCR of the treatment group were 78.1% and 92.7%, respectively, while those of the control group were 66.4% and 79.9%, besides, which significantly reduced the incidence of neurotoxicity and other toxic and side effects (52.2% vs. 94.6%) and prolonged OS about 2.7 months (Diao et al., 2018). The ORR and DCR in the treatment group of Weifu formula combined with FOLFOX6 were significantly improved (ORR, 70.0% vs. 26.7%; DCR, 83.3% vs. 56.7%), and the KPS score was also improved (83.3% vs. 60.0%) (Fan et al., 2015). However, these individual studies are not enough to explain the clinical role of traditional Chinese medicine (TCM) in AGC. Evidence-based medicine should be used to comprehensively analyze existing clinical studies and to evaluate available evidence. Due to the complexity and diversity of Chinese medicine prescriptions, it is impossible to determine which CHM herbs play an important role to synergistic action with chemotherapy.

With the further development of basic research, the role and mechanism of TCM in inhibiting the division and proliferation of tumor cells, promoting the apoptosis of tumor cells, inhibiting the metastasis of tumor cells, and enhancing the curative effect with chemotherapy have been gradually revealed, laying a foundation for the effective application of anti-tumor (Zhou et al., 2016; Liu et al., 2020; Ren et al., 2020; Xiang et al., 2020; Zhang L. et al., 2021). Available experimental studies indicate that TCM compounds do have anti-tumor activity, but the more appropriate and accurate drug selection is still unknown. Therefore, future research will focus on finding individual herbs with special contributions to chemotherapy efficacy of AGC, and to improve survival benefit of AGC and precise selection of TCM.

There are several RCTs evaluated the efficacy and safety of CHM in AGC, but these clinical evidence were not systematically evaluated. Furthermore, whether CHM have a synergistic effect with oxaliplatin-based chemotherapy, the key component of the first-line treatment of AGC, also need further evaluation. The objective of this study was to systematically evaluate the available evidence of tumor response and safety of CHM combined with oxaliplatin-based chemotherapy in AGC. Furthermore, we performed sensitivity analysis of single herb and combination of herbs, to explore the potential anti-tumor effects of these herbs.

## 2 Methods

This study was performed by the guidance of the Preferred Reporting Items for Systematic Reviews and Meta-Analyses (PRISMA) statement and checklist (Moher et al., 2009), PRISMA checklist is available in Supplementary Material S1. This study was registered on PROSPERO (No. CRD42022262595).

### 2.1 Eligibility criteria

#### 2.1.1 Type of studies

This study included RCTs with or without the blinded method, observational studies and quasi-RCTs were excluded. Trials did not describe the randomization process in details were considered as non-RCTs, and were excluded. Animal studies were also excluded.

#### 2.1.2 Types of participants

RCTs which participants diagnosed with AGC through cytological or pathological tests were included.

#### 2.1.3 Types of intervention and control

The intervention of CHM combined with oxaliplatin-based chemotherapy, and control of oxaliplatin-based chemotherapy were included in this study. We only included CHM formulas or oral patented drugs as the treatment of intervention, Chinese medicine injections and plant extracts were excluded.

#### 2.1.4 Types of outcomes

RCTs reporting outcomes of tumor response and safety of CHM in GC treatment were included in this study. Trials reported other efficacy outcomes were excluded. Given the strong correlation between the two anti-tumor treatment response evaluation criteria, WHO criteria, and RECIST criteria, the outcomes reported by these two criteria were considered homogeneous (Aras et al., 2016).

### 2.2 Search strategy

We searched PubMed, EMBASE, CENTRAL, Web of Science, the Chinese Biomedical Literature Database (CBM), the China National Knowledge Infrastructure (CNKI), the Wanfang database, and the Chinese Scientific Journals Database (VIP database). Searches were performed from the databases initiation to April 2022. The language restriction was English and Chinese. The search strategy was based on the combination of controlled vocabulary (MeSH terms and Emtree terms) and free-text terms. The terms of “Stomach Neoplasms,” “Oxaliplatin,” “Antineoplastic Combined Chemotherapy Protocols,” “Herbal Medicine,” “Medicine, Chinese Traditional,” and “Drugs, Chinese Herbal” were used to develop the search strategy for PubMed, which is shown in Supplementary Material S2. Modifications to the search strategy were used with other databases.

### 2.3 Screening and selection

Search results were imported to EndNote 20. The titles and abstracts of retrievals were screened after duplicates removal, then full articles of potential trials were assessed for their eligibility. Screening and selection were independently and in duplicate performed by the review authors (YT and HW). RCTs that met the inclusion criteria were included. The process was summarized using a PRISMA flow diagram.

### 2.4 Data extraction

The following data were extracted from the included studies: 1) identification information (first author, year of publication); 2) general information (study setting, sample size, and duration of follow-up); 3) participants (clinical stage, age, and sex); 4) intervention details (name of CHM intervention, compositions, and duration); 5) comparison details (chemotherapy regimen, dose, frequency, and duration of treatment), and 6) outcomes details.

### 2.5 Quality assessment

The Risk of Bias 2 (RoB-2) tool was used to assess the methodological quality of included studies (Sterne et al., 2019). We evaluated included studies of quality of the randomization process, deviation from intended intervention, missing outcome data, outcome measurement, and selection of the reported result. The overall quality of RCTs were evaluated as low, some concerns or high RoB.

### 2.6 Evidence synthesis for RCTs

Stata 16 was used in data synthesis to perform a meta-analysis. The RR for dichotomous data with 95% CIs were evaluated. The random-effects model was used when synthesizing data for the meta-analysis. As for the outcomes reported with zero event, the Mantel-Haenszel methods were adopted. We quantified inconsistency by applying the I^2^ statistic; a value of I^2^ > 40% was considered important heterogeneity, and I^2^ > 75% was considerable heterogeneity (Higgins et al., 2019). Subgroup analysis were performed according to the different regimens of chemotherapy that patients received, and to explore the source of heterogeneity if substantial heterogeneity existed. Publication bias of the cumulative evidence among individual studies was evaluated using a graphical method of funnel plot (Egger et al., 1997).

### 2.7 Sensitivity analysis

We performed sensitivity analysis to investigate the potential contributions of specific herbs to tumor response. Previous studies proposed that if a particular herb possessed anti-tumor effects, they would be reflected in the pooled effect estimates of the studies which interventions containing this herb (Chen et al., 2016a; Chen et al., 2016b). Sensitivity analysis of ORR will be performed for studies on herbs used in AGC, herbs, or combinations of herbs presented in two or more studies, and the following principles will be applied:

1) Studies containing the same herb or combination of herbs will be treated as one, and the pooled RR (95% CI) and I^2^ will be calculated; 2) herbs or combinations of herbs will be excluded if there is no significant effect in the pooled results (95% CIs of RR overlap 1.0) and/or important heterogeneity exists between studies (I^2^ ≥ 40%); 3) the RR results will be listed in ascending order with 95% CI, the number of studies and I^2^ values; 4) the combination of herbs will be excluded when they have lower RRs than herbs alone; and 5) when herb combinations have higher RRs than herbs alone, they will be identified as potential examples of synergistic effects.

### 2.8 Quality of evidence

The quality of the cumulative evidence was evaluated using the Grading of Recommendations Assessment, Development, and Evaluation (GRADE) system. Study limitations, inconsistency, indirectness, imprecision, and publication bias were evaluated. Quality of evidence was classified as high, moderate, low, or very low quality (Guyatt et al., 2008). We presented our findings in a Summary of Finding table.

## 3 Results

**FIGURE 1:**
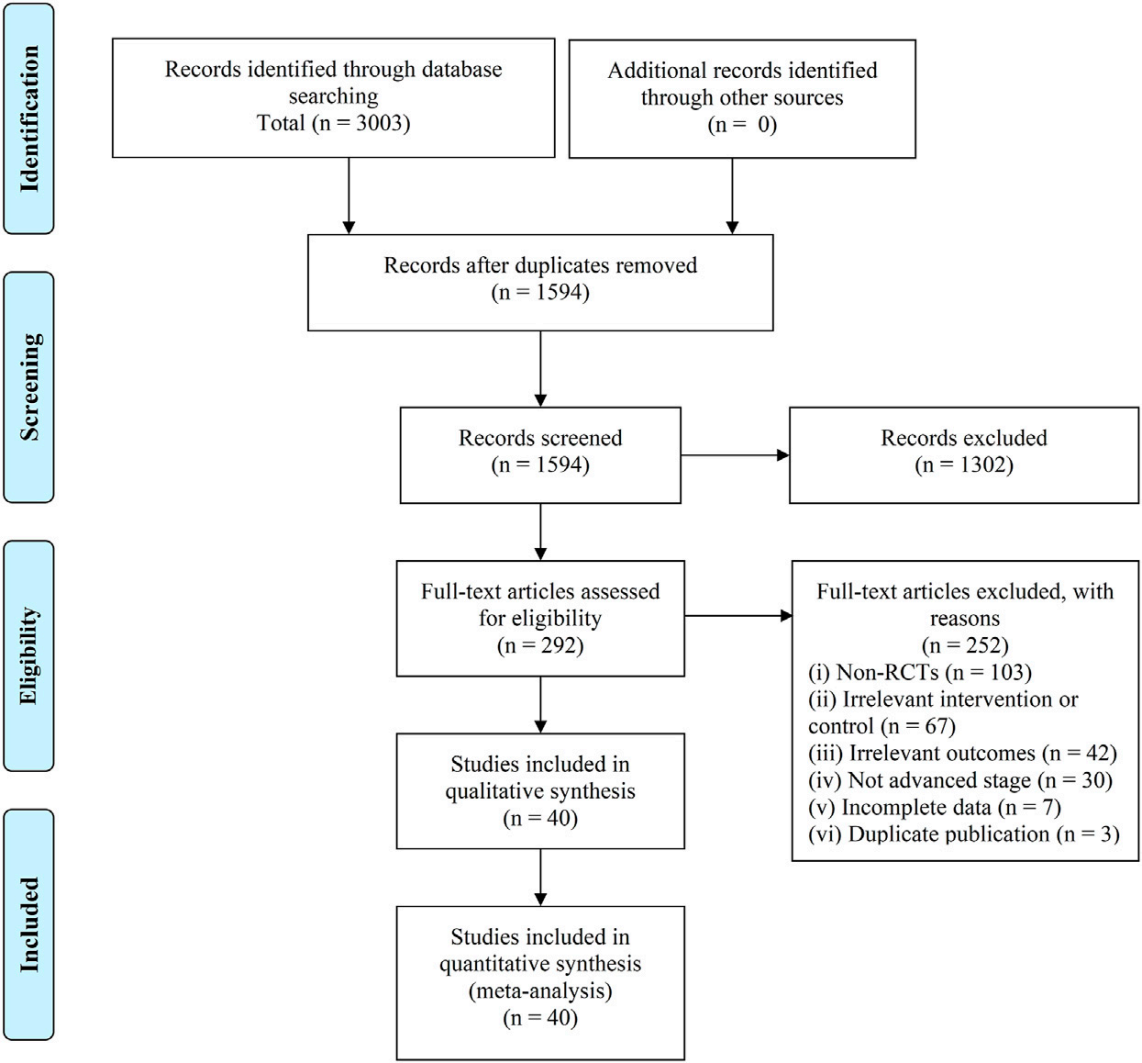
Flowchart of records screening and selection.

There were 3,003 retrievals exported from databases searches, and after the selection process, 40 trials involving 3,029 participants were included in this SR (Chen et al., 2007; Chi et al., 2010; Hu, 2011; Yuan, 2011; Zhao, 2011; Zhu et al., 2011; Qin, 2012; Guo, 2014; Huang, 2014; Liu et al., 2015; Li et al., 2016; Zhao et al., 2016; Chu et al., 2017; Feng, 2017; Huang et al., 2017; Wang N. et al., 2018; Yang et al., 2018; Yuan, 2018; Cai et al., 2019; Gu et al., 2019; Jiao, 2019; Liu et al., 2019; Xie, 2019; Yu et al., 2019; Zhai, 2019; Zhang, 2019; Zhong et al., 2019; Bao, 2020; Gong, 2020; Li D. H. et al., 2020; Sun et al., 2020; Zhang et al., 2020; Zhang, 2021a; Zhang, 2021b; Feng et al., 2021; Jiang et al., 2021; Long, 2021; Zhang H. O. et al., 2021; Zhao, 2021; Zhong et al., 2021). The selection process was summarized as a flowchart shown in Figure 1.

### 3.1 Details of included trials

All these 40 trials are non-blinded RCTs that conducted in single-center. The sample sizes of included trials ranged from 50 to 124. Among these included trials, one published in an English journal (Li Y. et al., 2020), and other 39 trials were published in Chinese (Chen et al., 2007; Chi et al., 2010; Hu, 2011; Yuan, 2011; Zhao, 2011; Zhu et al., 2011; Qin, 2012; Guo, 2014; Huang, 2014; Liu et al., 2015; Li et al., 2016; Zhao et al., 2016; Chu et al., 2017; Feng, 2017; Huang et al., 2017; Wang Y. et al., 2018; Yang et al., 2018; Yuan, 2018; Cai et al., 2019; Gu et al., 2019; Jiao, 2019; Liu et al., 2019; Xie, 2019; Yu et al., 2019; Zhai, 2019; Zhang, 2019; Zhong et al., 2019; Bao, 2020; Gong, 2020; Sun et al., 2020; Zhang et al., 2020; Zhang, 2021a; Zhang, 2021b; Feng et al., 2021; Jiang et al., 2021; Long, 2021; Zhang L. et al., 2021; Zhao, 2021; Zhong et al., 2021). The characteristics of included RCTs were shown in Table 1.

**TABLE 1:** Study characteristics of included RCTs.

Study	Study Design	Sample Size		Age		Sex (Male/Female)		Stage		Outcomes
		T	C	T	C	T	C	T	C	
Bao (2020)	Single center	34	34	44–86 (66.42 ± 7.48)	45–87 (65.73 ± 7.21)	21/13	22/12	III-IV	III-IV	①②③
Cai et al. (2019)	Single center	60	60	42–74 (61.5 ± 7.9)	41–73 (61.2 ± 7.6)	35/25	38/22	III-IV	III-IV	①②③
Chen et al. (2007)	Single center	27	23	34–78 (median: 58)		32/18		IV	IV	①③
Chi et al. (2010)	Single center	30	30	40–70 (median: 57)		38/22		IV	IV	①②③
Chu et al. (2017)	Single center	30	30	42–68 (56.3 ± 4.2)	40–75 (55.6 ± 4.5)	20/10	22/8	IIIb-IV	IIIb-IV	①②③
Feng et al. (2021)	Single center	41	41	35–67 (52.29 ± 4.71)	34–69 (53.84 ± 4.91)	23/18	21/20	III-Ⅳ	III-Ⅳ	①③
Feng (2017)	Single center	30	30	>60: 19, ≤60: 11	>60: 21, ≤60: 9	18/12	22/8	III-IV	III-IV	①②③
Gong (2020)	Single center	32	32	66.08 ± 9.52	(65.56 ± 7.65	19/13	17/15	III-IV	III-IV	①③
Gu et al. (2019)	Single center	35	35	27–76 (48.3 ± 5.2)	28–77 (47.5 ± 4.8)	19/16	21/14	III-IV	III-IV	①
Guo (2014)	Single center	30	30	57.10 ± 6.65	56.26 ± 7.51	22/8	21/9	IIIb-IV	IIIb-IV	①②③
Hu et al. (2012)	Single center	51	48	35–74 (57.65 ± 9.42)	32–75 (59.09 ± 10.62)	35/16	34/14	IV	IV	①②③
Huang et al. (2017)	Single center	34	34	41–70 (57.9 ± 61.2)	40–69 (58.9 ± 6.6)	19/15	20/14	III-IV	III-IV	①②③
Huang (2014)	Single center	32	32	55.13 ± 10.676	53.97 ± 10.304	17/15	16/16	IV	IV	①②③
Jiang et al. (2021)	Single center	51	51	55.78 ± 6.85	55.92 ± 6.49	32/19	30/21	III-IV	III-IV	①③
Jiao (2019)	Single center	52	52	32–68 (54.23 ± 8.67)	33–70 (56.32 ± 8.40)	27/25	29/23	IV	IV	①
Li et al. (2016)	Single center	34	34	46.35 ± 6.21	46.97 ± 6.31	20/14	22/12	T3-T4	T3-T4	①②③
Li D. H. et al. (2020)	Single center	29	28	45–73 (57.36 ± 8.87)	44–75 (58.25 ± 9.64)	17/12	16/12	IIIb-IV	IIIb-IV	①②③
Liu et al. (2015)	Single center	62	62	61.8 ± 11.6	60.5 ± 10.8	43/19	46/16	IIIb-IV	IIIb-IV	①②③
Liu et al. (2019)	Single center	48	48	54.37 ± 8.10	55.09 ± 8.05	26/22	25/23	IV	IV	①②③
Long (2021)	Single center	32	32	59.03 ± 8.32	57.81 ± 7.49	23/19	20/12	IV	IV	②③
Qin (2012)	Single center	27	26	59.04 ± 8.716	57.73 ± 10.724	19/8	20/6	III-IV	III-IV	①②③
Sun et al. (2020)	Single center	40	40	35–74 (63.1 ± 8.6)	32–75 (2.7 ± 8.9)	27/13	26/14	III-IV	III-IV	①②③
Wang N. et al. (2018)	Single center	60	60	55–78 (64.16 ± 1.17)	61–79 (68/58 ± 1.25)	34/26	31/29	III-IV	III-IV	①②③
Xie (2019)	Single center	33	31	62.73 ± 8.769	59.52 ± 9.095	18/15	15/10	III-IV	III-IV	①②③
Yang et al. (2018)	Single center	40	40	59.41 ± 13.59	59.41 ± 13.59	21/19	23/17	III-IV	III-IV	①②③
Yu et al. (2019)	Single center	41	41	42–78 (55.17 ± 5.86)	43–79 (56.30 ± 6.28)	23/18	25/16	IIIb-IV	IIIb-IV	①②③
Yuan (2011)	Single center	26	25	18–75	18–75	18/8	20/5	IV	IV	①②③
Yuan (2018)	Single center	30	30	58.93 ± 7.056	61.43 ± 7.142	23/7	20/10	IIIb-IV	IIIb-IV	①②③
Zhai (2019)	Single center	32	32	18–70	18–70	24/8	20/12	IIIb	IIIb	①②③
Zhang H. O. et al. (2021)	Single center	30	30	66.66 ± 5.912	67.21 ± 6.425	21/9	21/9	IIIb-IV	IIIb-IV	①②③
Zhang H. O. et al. (2021)	Single center	30	30	62.16 ± 9.53	64.30 ± 8.92	13/17	11/19	III-IV	III-IV	①②③
Zhang L. et al. (2021)	Single center	28	27	65.57 ± 6.06	66.56 ± 5.57	20/8	18/9	IIIb-IV	IIIb-IV	①②③
Zhang et al. (2020)	Single center	43	43	33–80 (50.41 ± 8.16)	35–78 (49.06 ± 7.34)	24/19	26/17	III-IV	III-IV	①③
Zhang (2019)	Single center	50	50	65.24 ± 5.27	68.46 ± 5.94	30/16	26/11	III-IV	III-IV	①②③
Zhao (2011)	Single center	30	24	57.60 ± 11.34	57.47 ± 12.06	19/11	11/7	IIIb-IV	IIIb-IV	①②③
Zhao et al. (2016)	Single center	39	39	38–70 (57.03 ± 9.47)	41–69 (58.31 ± 10.23)	28/11	26/13	III-IV	III-IV	①②③
Zhao (2021)	Single center	49	49	48–77 (57.21 ± 4.58)	47–76 (56.87 ± 4.62)	26/23	25/24	IIIb-IV	IIIb-IV	①③
Zhong et al. (2021)	Single center	41	41	36.70 (58.8 ± 9.2)	35.70 (58.3 ± 9.5)	24/17	22/19	IIIb-IV	IIIb-IV	①③
Zhong et al. (2019)	Single center	41	41	33–76 (54.86 ± 3.77)	32–78 (55.04 ± 3.14)	22/19	20/21	IV	IV	①②③
Zhu et al. (2011)	Single center	40	40	35–86 (54.5 ± 4.5)		51/29		III-IV	III-IV	①②

T Treatment group, C Control group.

Outcomes: ①Tumor Response, ②Quality of Life, ③Adverse Events.

#### 3.1.1 Intervention details

Among these 40 trials, three trials adopted TCM syndrome differentiation treatment, and patients in these three trials received more than one core prescription, other 37 trials used single formula as core prescription. As for the chemotherapy, SOX was the most frequent regimen that adopted by 16 trials (Qin, 2012; Guo, 2014; Liu et al., 2015; Zhao et al., 2016; Feng, 2017; Wang N. et al., 2018; Yuan, 2018; Zhai, 2019; Zhang, 2019; Bao, 2020; Gong, 2020; Li Y. et al., 2020; Zhang et al., 2020; Zhang, 2021a; Jiang et al., 2021; Zhao, 2021), FOLFOX regimen was adopted in 14 trials (Chen et al., 2007; Chi et al., 2010; Zhao, 2011; Zhu et al., 2011; Huang, 2014; Li et al., 2016; Chu et al., 2017; Huang et al., 2017; Gu et al., 2019; Jiao, 2019; Liu et al., 2019; Zhong et al., 2019; Sun et al., 2020; Long, 2021), and XELOX regimen in 10 trials (Hu, 2011; Yuan, 2011; Yang et al., 2018; Cai et al., 2019; Xie, 2019; Yu et al., 2019; Zhang, 2021b; Feng et al., 2021; Zhang H. O. et al., 2021; Zhong et al., 2021). Intervention details of included RCTs were shown in Table 2. The Chinese phonetic transcription, scientific name, Latin drug name, and English name of herbs adopted in the prescriptions of included trials were shown in Table 3.

**TABLE 2:** Intervention details of included RCTs.

Study	Treatment in intervention group	Treatment in intervention group
Bao (2020)	Yiqi Huoxue Formula combined with SOX regimen chemotherapy. Formula composition: Acruginous turmeric, Chinese angelica, Finger citron, Villous amomum, Sanchi, Codonopsis, Astragalus	SOX regimen for 4 cycles: S-1 Capsules 40 mg, twice daily, taking it for 14 days, stopping for 7 days; Oxaliplatin injection 130 mg/m^2^ intra venous drip for 3 h on day 1, one cycle for 21 days
Cai et al. (2019)	Jianpi Yiqi Formula combined with XELOX regimen chemotherapy. Formula composition: Codonopsis, Poria, Largehead atractylodes, Perilla frutescens leaf, Coxi seed, Pinellia, Inula, Ruddle, Villous amomum, Liquorice	XELOX regimen for 3 cycles: Capetabine 1,000 mg/m^2^, twice daily, taking it for 14 days, stopping for 7 days; Oxaliplatin injection 130 mg/m^2^ intra venous drip on day 1, one cycle for 21 days
Chen et al. (2007)	TCM syndrome differentiation treatment combined with FOLFOX regimen chemotherapy	FOLFOX regimen chemotherapy: Oxaliplatin injection 130 mg/m^2^ intra venous drip on day 1; CF 200 mg intra venous drip on day 1–3; 5-Fu 400 mg/m^2^ on day 1, and a infusion (2,000 mg/m^2^) for 70 consecutive hours, one cycle for 21 days
Chi et al. (2010)	Yiqi Huoxue Formula combined with FOLFOX regimen chemotherapy. Formula composition: Astragalus, Pseudostellaria, Suberect spatholobus, Largehead atractylodes, Poria, Wolfberry, Ligustrum, Cuscuta, Red paeony, Coxi seed, Actinidia root	FOLFOX4 regimen chemotherapy: Oxaliplatin injection 85 mg/m^2^ intra venous drip on day 1; CF 200 mg/m^2^ intra venous drip on day 1–2; 5-Fu 400 mg/m^2^ on day 1–2, and a 20-h infusion (600 mg/m^2^/d) for 2 consecutive days, one cycle for 21 days
Chu et al. (2017)	Zhangshi Yiwei Decoction combined with FOLFOX regimen chemotherapy. Formula composition: Largehead atractylodes, Poria, Codonopsis, Coptis, Tangerine peel, Thunberbg fritillary, Aucklandia, Pinellia, Villous amomum, Dandelion, Silktree bark, Bletilla, Liquorice	FOLFOX4 regimen chemotherapy: Oxaliplatin injection 130 mg/m^2^ intra venous drip on day 1; CF 100 mg/m^2^ intra venous drip on day 1–5; 5-Fu 300 mg/m^2^ on day 1–5, one cycle for 21 days
Feng et al. (2021)	Xuezheng Decoction combined with XELOX regimen chemotherapy. Formula composition: Acruginous turmeric, Hedyotis diffusa, Smilax, Chinese angelica, Red paeony, Sparganii, Cassia twig, Frankincense, Myrrh	XELOX regimen for 2 cycles: Capetabine 1,000 mg/m^2^, twice daily, once after breakfast and dinner respectively, taking it for 14 days, stopping for 7 days; Oxaliplatin injection 85 mg/m^2^ intra venous drip on day 1, one cycle for 21 days
Feng (2017)	Gancao Xiexin Decoction combined with SOX regimen chemotherapy. Formula composition: Liquorice, Scutellaria, Coptis, Pinellia, Dried ginger, Ginseng, Red date	SOX regimen: S-1 Capsules 40–60 mg, twice daily, once after breakfast and dinner respectively, taking it for 14 days, stopping for 7 days; Oxaliplatin injection 130 mg/m^2^ intra venous drip for 3 h on day 1, one cycle for 21 days
Gong (2020)	Wenyang Sanjie Decoction combined with SOX regimen chemotherapy. Formula composition: Astragalus, Yam, Chinese clematis, Coxi seed, Scutellaria barbata, White paeony, Ruddle, Largehead atractylodes, Psoralea, Cuscuta, Barley sprout, Dandelion, Wolfberry, Acruginous turmeric, Membrane of chickens gizzard, Inula, Pinellia, Gekko, Cassia twig, Tangerine peel, Dried ginger	SOX regimen: S-1 Capsules 40–60 mg, twice daily, once after breakfast and dinner respectively, taking it for 14 days, stopping for 7 days; Oxaliplatin injection 130 mg/m^2^ intra venous drip for 3 h on day 1, one cycle for 21 days
Gu et al. (2019)	Jianwei Yiai Powder combined with FOLFOX regimen chemotherapy. Formula composition: Nightshade, Vietnamese sophora root, Scutellaria barbata, Hedyotis diffusa, Scolopendra, Pinellia, Arcae concha, Aucklandia, Poria, Suberect spatholobus, Codonopsis, Largehead atractylodes, Liquorice	FOLFOX4 regimen chemotherapy: Oxaliplatin injection 85 mg/m^2^ intra venous drip on day 1; CF 200 mg/m^2^ intra venous drip on day 1–2; 5-Fu 400 mg/m^2^ on day 1, and a 22-h infusion (600 mg/m^2^/d) for 2 consecutive days, one cycle for 21 days
Guo (2014)	Wenyang Sanjie Decoction combined with SOX regimen chemotherapy. Formula composition: Codonopsis, Astragalus, Largehead atractylodes, Poria, Ligustrum, Pinellia, Hedyotis diffusa, Cremastra, Tangerine peel, Actinidia root, Membrane of chickens gizzard, Liquorice	SOX regimen: S-1 Capsules 40 mg, twice daily, once after breakfast and dinner respectively, taking it for 14 days, stopping for 7 days; Oxaliplatin injection 130 mg/m^2^ intra venous drip for 3 h on day 1, one cycle for 21 days
Hu (2011)	Yiqi Huoxue Jiedu Formula combined with XELOX regimen chemotherapy. Formula composition: Astragalus, Codonopsis, Pseudostellaria, Largehead atractylodes, Poria, Wolfberry, Ligustrum Cuscuta, Suberect spatholobus, Red paeony, Acruginous turmeric, Paris polyphylla, Hedyotis diffusa, Actinidia root	XELOX regimen for 3 cycles: Capetabine 1,000 mg/m^2^, twice daily, once after breakfast and dinner respectively, taking it for 14 days, stopping for 7 days; Oxaliplatin injection 85 mg/m^2^ intra venous drip on day 1, one cycle for 21 days
Huang (2017)	Jianpi Yangwei Formula combined with FOLFOX4 regimen chemotherapy. Formula composition: Hedyotis chrysotricha, Smilax, Coxi seed, Yam, Codonopsis, Largehead atractylodes, Poria, Aucklandia, Chinese angelica, White paeony, Liquorice	FOLFOX4 regimen chemotherapy: Oxaliplatin injection 85 mg/m^2^ intra venous drip on day 1; CF 200 mg/m^2^ intra venous drip on day 1–2; 5-Fu 400 mg/m^2^ on day 1, and a 22-h infusion (600 mg/m^2^/d) for 2 consecutive days, one cycle for 21 days
Huang (2014)	Jianpi Huayu Decoction combined with FOLFOX4 regimen chemotherapy. Formula composition: Codonopsis, Largehead atractylodes, Dandelion, Perilla frutescens stem, Nardostachys, Snakegourd seed, Pinellia, Gekko, Acruginous turmeric, Crataegi, Rhei, Magnolia bark, Membrane of chickens gizzard	FOLFOX4 regimen chemotherapy: Oxaliplatin injection 85 mg/m^2^ intra venous drip on day 1; CF 200 mg/m^2^ intra venous drip on day 1–2; 5-Fu 400 mg/m^2^ on day 1, and a 22-h infusion (600 mg/m^2^/d) for 2 consecutive days, one cycle for 21 days
Jiang et al. (2021)	Jiedu Sanjie Formula combined with SOX regimen chemotherapy. Formula composition: Dandelion, Prunella, Honeysuckle, Forsythia, Chinese angelica, Figwort, Isatis, Stiff silkworm, Myrrh, Scorpion, Gleditsia sinensis	SOX regimen: S-1 Capsules 40–60 mg, twice daily, taking it for 14 days, stopping for 7 days; Oxaliplatin injection 130 mg/m^2^ intra venous drip for 3 h on day 1, one cycle for 21 days
Jiao (2019)	Modified Xuezheng Decoction combined with FOLFOX4 regimen chemotherapy. Formula composition: Sparganii, Acruginous turmeric, Hedyotis diffusa, Smilax, Chinese angelica, Red paeony, Frankincense, Myrrh, Cassia twig	FOLFOX4 regimen chemotherapy: Oxaliplatin injection 85 mg/m^2^ intra venous drip on day 1; CF 200 mg/m^2^ intra venous drip on day 1–2; 5-Fu 400 mg/m^2^ on day 1, and a 22-h infusion (600 mg/m^2^/d) for 2 consecutive days, one cycle for 21 days
Li (2016)	Fuzheng Kangai Formula combined with FOLFOX4 regimen chemotherapy. Formula composition: Largehead atractylodes, Morinda, Wolfberry, Drynaria Rehmannia glutinosa, Epimedium, Cornus, Ginseng, Eucommia, Psoralea, Cassia bark, Chinese angelica, Curculigo	FOLFOX4 regimen chemotherapy: Oxaliplatin injection 85 mg/m^2^ intra venous drip on day 1; CF 200 mg/m^2^ intra venous drip on day 1–2; 5-Fu 400 mg/m2 on day 1, and a 22-h infusion (600 mg/m^2^/d) for 2 consecutive days, one cycle for 21 days
Li Y. et al. (2020)	Shunqi Yiwei Decoction combined with SOX regimen chemotherapy. Formula composition: Bupleurum, Aurantii fructus, White paeony, Liquorice, Poria, Atractylodis, Codonopsis	SOX regimen: S-1 Capsules 40 mg, twice daily, once after breakfast and dinner respectively, taking it for 14 days, stopping for 7 days; Oxaliplatin injection 130 mg/m^2^ intra venous drip for 3 h on day 1, one cycle for 21 days
Liu et al. (2015)	Jianpi Xiaozheng Formula combined with SOX regimen chemotherapy. Formula composition: Astragalus, Smilax, Hedyotis chrysotricha, Coxi seed, Codonopsis, Poria, Yam, Sparganii, Acruginous turmeric, Largehead atractylodes, Aucklandia, Tangerine peel, Chinese angelica, White paeony	SOX regimen: S-1 Capsules 40–60 mg, twice daily, taking it for 14 days, stopping for 7 days; Oxaliplatin injection 130 mg/m^2^ intra venous drip for 3 h on day 1, one cycle for 21 days
Liu et al. (2019)	Jianpi Huayu Formula combined with FOLFOX4 regimen chemotherapy. Formula composition: Largehead atractylodes, Dandelion, Perilla frutescens stem, Nardostachys, Snakegourd seed, Pinellia, Gekko, Acruginous turmeric, Crataegi, Rhei, Magnolia bark, Membrane of chickens gizzard	FOLFOX4 regimen chemotherapy: Oxaliplatin injection 85 mg/m^2^ intra venous drip on day 1; CF 200 mg/m^2^ intra venous drip on day 1–2; 5-Fu 400 mg/m^2^ on day 1, and a 22-h infusion (600 mg/m^2^/d) for 2 consecutive days, one cycle for 21 days
Long (2021)	Yiqi Fuyuan Formula combined with mFOLFOX6 regimen chemotherapy. Formula composition: Astragalus, Codonopsis, Largehead atractylodes, Aucklandia, Tangerine peel, Poria, Chinese angelica, Spine date seed, Yam, Raw ginger, Lotus seed, Liquorice	mFOLFOX6 regimen chemotherapy: Oxaliplatin injection 85 mg/m^2^ intra venous drip on day 1; CF 400 mg/m^2^ intra venous drip on day 1; 5-Fu 400 mg/m^2^ on day 1, and a infusion (2,400 mg/m^2^) for 46 consecutive hours, one cycle for 21 days
Qin (2012)	Modified Buzhong Yiqi Decoction combined with SOX regimen chemotherapy. Formula composition: Astragalus, Codonopsis, Largehead atractylodes, Liquorice, Chinese angelica, Sichuan lovase, Tangerine peel, Acruginous turmeric, Hedyotis diffusa, Coxi seed	SOX regimen: S-1 Capsules 80 mg, twice daily, taking it for 14 days, stopping for 7 days; Oxaliplatin injection 130 mg/m^2^ intra venous drip for 3 h on day 1, one cycle for 21 days
Sun et al. (2020)	Fuzheng Kangai Formula combined with FOLFOX6 regimen chemotherapy. Formula composition: Codonopsis, Largehead atractylodes, Bupleurum, White paeony, Poria, Tangerine peel, Pinellia, Sichuan lovase, Aurantii fructus, Ligustrum, Ganoderma, Barley sprout, Millet sprout, Paris polyphylla, Scutellaria barbata, Hedyotis diffusa, Liquorice	FOLFOX6 regimen chemotherapy: Oxaliplatin injection 85 mg/m^2^ intra venous drip on day 1; CF 400 mg/m^2^ intra venous drip on day 1; 5-Fu 400 mg/m^2^ on day 1, and a infusion (2,400 mg/m^2^) for 46 consecutive hours, one cycle for 21 days
Wang Y. et al. (2018)	Guishao Liujunzi Decoction combined with SOX regimen chemotherapy. Formula composition: Chinese angelica, White paeony, Codonopsis, Poria, Largehead atractylodes, Yam, Coxi seed, Tangerine peel, Pinellia, Hedyotis diffusa, Scutellaria barbata, Sparganii, Acruginous turmeric, Liquorice	SOX regimen: S-1 Capsules 50 mg, twice daily, taking it for 14 days, stopping for 7 days; Oxaliplatin injection 130 mg/m^2^ intra venous drip on day 1, one cycle for 21 days
Xie (2019)	Wendan Decoction combined with XELOX regimen chemotherapy. Formula composition: Pinellia, Bambusae caulis, Aurantii fructus Immaturus, Tangerine peel, Poria, Liquorice, Raw ginger, Red date	XELOX regimen for 3 cycles: Capetabine 1,000 mg/m^2^, twice daily, once after breakfast and dinner respectively, taking it for 14 days, stopping for 7 days; Oxaliplatin injection 85 mg/m^2^ intra venous drip on day 1, one cycle for 21 days
Yang et al. (2018)	Shenyu Yangwei Decoction combined with XELOX regimen chemotherapy. Formula composition: Ginseng, Cornus, Dendrobium, Salviae	XELOX regimen for 2 cycles: Capetabine 1,000 mg/m^2^, twice daily, once after breakfast and dinner respectively, taking it for 14 days, stopping for 7 days; Oxaliplatin injection 130 mg/m^2^ intra venous drip on day 1, one cycle for 21 days
Yu et al. (2019)	Modified Shengyang Yiwei Decoction combined with XELOX regimen chemotherapy. Formula composition: Ginseng, Cornus, Dendrobium, Salviae	XELOX regimen for 2 cycles: Capetabine 1,000 mg/m^2^, twice daily, once after breakfast and dinner respectively, taking it for 14 days, stopping for 7 days; Oxaliplatin injection 130 mg/m^2^ intra venous drip on day 1, one cycle for 21 days
Yuan (2011)	Modified Shengyang Yiwei Decoction combined with XELOX regimen chemotherapy. Formula composition: Codonopsis, Largehead atractylodes, Poria, Coxi seed, Pinellia, Tangerine peel, Agaric Yam, Millet sprout, Barley sprout, Poria with hostwood, Loquat leaf, Liquorice	XELOX regimen for 2 cycles: Capetabine 1,000 mg/m^2^, twice daily, once after breakfast and dinner respectively, taking it for 14 days, stopping for 7 days; Oxaliplatin injection 130 mg/m^2^ intra venous drip for 2 h on day 1, one cycle for 21 days
Yuan (2018)	Yiqi Jiedu Formula combined with SOX regimen chemotherapy. Formula composition: Ginseng, Gekko, Paris polyphylla, Pinellia, Tangerine peel, Magnolia bark, Liquorice	SOX regimen: S-1 Capsules 40 mg, twice daily, taking it for 14 days, stopping for 7 days; Oxaliplatin injection 130 mg/m^2^ intra venous drip on day 1, one cycle for 21 days
Zhai (2019)	Huayu Jiedu Formula combined with SOX regimen chemotherapy. Formula composition: Scorpion, Gekko, Sanchi, Nitroum, Scutellaria barbata, Membrane of chickens gizzard	SOX regimen: S-1 Capsules 40–60 mg, twice daily, once after breakfast and dinner respectively, taking it for 14 days, stopping for 7 days; Oxaliplatin injection 130 mg/m^2^ intra venous drip for 3 h on day 1, one cycle for 21 days
Zhang (2021a)	Modified Shiquan Dabu Decoction combined with XELOX regimen chemotherapy. Formula composition: Astragalus, Coxi seed, Largehead atractylodes, Poria, Chinese angelica, Pinellia, White paeony, Rehmannia glutinosa, Sparganii, Acruginous turmeric, Sichuan lovase, Ginseng, Cassia bark, Villous amomum, Liquorice	XELOX regimen: Capetabine 1,000 mg/m^2^, twice daily, once after breakfast and dinner respectively, taking it for 14 days, stopping for 7 days; Oxaliplatin injection 130 mg/m^2^ intra venous drip for 2 h on day 1, one cycle for 21 days
Zhang (2021a)	TCM syndrome differentiation treatment combined with XELOX regimen chemotherapy	XELOX regimen for 3 cycles: Capetabine 1,000 mg/m^2^, twice daily, once after breakfast and dinner respectively, taking it for 14 days, stopping for 7 days; Oxaliplatin injection 130 mg/m^2^ intra venous drip for 2 h on day 1, one cycle for 21 days
Zhang (2021b)	Shugan Yangwei Decoction combined with SOX regimen chemotherapy. Formula composition: Bupleurum, White paeony, Scutellaria, Aurantii fructus Immaturus, Coptis, Dried ginger, Pinellia, Aucklandia, Rhei, Salviae, Toosendan Corydalis Codonopsis, Katsumada galangal, Liquorice	SOX regimen: S-1 Capsules 40∼60 mg, twice daily, taking it for 14 days, stopping for 7 days; Oxaliplatin injection 130 mg/m^2^ intra venous drip on day 1, one cycle for 21 days
Zhang et al. (2020)	Shengyang Yiwei Decoction combined with SOX regimen chemotherapy. Formula composition: Astragalus, Pinellia, Ginseng, Liquorice, Angelicae tuhuo, Divaricate saposhniovia, White paeony, Notopterygium, Tangerine peel, Poria, Bupleurum, Alisma orientale, Largehead atractylodes, Coptis	SOX regimen: S-1 Capsules 60 mg, twice daily, taking it for 14 days, stopping for 7 days; Oxaliplatin injection 130 mg/m^2^ intra venous drip on day 1, one cycle for 21 days
Zhang (2019)	Erteng Sanjie Capsule combined with SOX regimen chemotherapy. Formula composition: Pseudostellaria, Largehead atractylodes, Coxi seed, Pinellia, Tangerine peel, Villous amomum, Gekko, Sargentgloryvine Smilax, Amur grape vines, Actinidia root, Prunella, Ostreae concha, Acruginous turmeric, Areca, Liquorice	SOX regimen: S-1 Capsules 40∼60 mg, twice daily, taking it for 14 days, stopping for 7 days; Oxaliplatin injection 130 mg/m^2^ intra venous drip on day 1, one cycle for 21 days
Zhao (2011)	Kun Shen Granule combined with FOLFOX regimen chemotherapy. Formula composition: Laminaria, Actinidia root, Agrimony, Ginseng	FOLFOX regimen chemotherapy for 2 cycles: Oxaliplatin injection 200 mg intra venous drip on day 1; CF 300 mg intra venous drip on day 1–5; 5-Fu 750 mg on day 1–5, one cycle for 21 days
Zhao (2016)	Jianpi Huayu Formula combined with SOX regimen chemotherapy. Formula composition: Pseudostellaria, Largehead atractylodes, Paris polyphylla, Salviae, Scutellaria barbata, Salvia chinensis, Hedyotis diffusa, Poria, Nightshade, Dendrobium, Yam	SOX regimen: S-1 Capsules 60∼80 mg, twice daily, taking it for 14 days, stopping for 7 days; Oxaliplatin injection 65 mg/m^2^ intra venous drip for 3 h on day 1 and day 8, one cycle for 21 days
Zhao (2021)	Wenyang Jianpi Decoction combined with SOX regimen chemotherapy. Formula composition: Pseudostellaria, Hedyotis diffusa, Largehead atractylodes, Acruginous turmeric, Nightshade, Millet sprout, Barley sprout, Pinellia, Poria, Tangerine peel, Aurantii fructus, Cassia twig, Dried ginger, Liquorice	SOX regimen: S-1 Capsules 40 mg, twice daily, taking it for 14 days, stopping for 7 days; Oxaliplatin injection 130 mg/m^2^ intra venous drip on day 1, one cycle for 21 days
Zhong et al. (2021)	Jianpi Fuzheng Xiaoliu Formula combined with XELOX regimen chemotherapy. Formula composition: Pseudostellaria, Coxi seed, Largehead atractylodes, Hedyotis diffusa, Smilax, Scutellaria barbata, Sparganii, Acruginous turmeric, Poria, Yam, Chinese angelica, Crataegi, Liquorice, Membrane of chickens gizzard	XELOX regimen for 4 cycles: Capetabine 1,000 mg/m^2^, twice daily, taking it for 14 days, stopping for 7 days; Oxaliplatin injection 130 mg/m^2^ intra venous drip for on day 1, one cycle for 21 days
Zhong et al. (2019)	Modified Shenling Baizhu Decoction combined with FOLFOX4 regimen chemotherapy. Formula composition: Tangerine peel, Cimicifuga, Bupleurum, Platycodon, Liquorice, Lotus seed, Villous amomum, Chinese angelica, Largehead atractylodes, Dolichos, Poria, Yam, Codonopsis, Astragalus, Coxi seed	FOLFOX4 regimen chemotherapy: Oxaliplatin injection 85 mg/m^2^ intra venous drip on day 1; CF 200 mg/m^2^ intra venous drip on day 1; 5-Fu 400 mg/m^2^ on day 1, and a 22-h infusion (600 mg/m^2^/d) for 2 consecutive days, one cycle for 21 days
Zhu (2011)	Jianpi Yiqi Decoction combined with FOLFOX regimen chemotherapy. Formula composition: Astragalus, Crataegi, Scorch-fried medicated leaven, Barley sprout, Adenophorae, Glehnia, Solomonseal, Tangerine peel, Pinellia, Finger citron, Magnolia bark, Membrane of chickens gizzard, Villous amomum, Whitefruit amomim, Liquorice	FOLFOX regimen chemotherapy for 2 cycles: Oxaliplatin injection 135 mg/m^2^ intra venous drip on day 1; CF 100 mg/m^2^ intra venous drip on day 1–5; 5-Fu 500 mg/m^2^ on day 1–5, one cycle for 21 days

**TABLE 3:** The names of Herbs.

Phonetic transcription	Scientific name	Latin drug name	English name
Bajitian	*Morinda officinalis* How	Morindae Officinalis Radix	Morinda
Baqia	*Smilax china* L.	Smilacis Chinae Rhizoma	Smilax
Baibiandou	*Dolichos lablab* L.	Lablab Semen Album	Dolichos
Baidoukou	*Amomum kravanh* Pierre ex Gagnep.	Amomi Fructus Rotundus	Whitefruit Amomim
Baihuasheshecao	*Hedyotis diffusa* Willd.	Hedyotis Diffusa Herba	Hedyotis diffusa
Baiji	*Bletilla striata* (Thunb.) Reichb.f.	Bletillae Rhizoma	Bletilla
Baishao	*Paeonia lactiflora* Pall.	Paeoniae Radix Alba	White Paeony
Baizhu	*Atractylodes macrocephala* Koidz.	Atractylodis Macrocephalae Rhizoma	Largehead Atractylodes
Banlangen	*Isatis indigotica* Fort.	Isatidis Radix	Isatis
Banxia	*Pinellia ternata* (Thunb.) Makino.	Pinelliae Rhizoma	Pinellia
Banzhilian	*Scutellaria barbata* D.Don	Scutellariae Barbatae Herba	Scutellaria barbata
Beishanshen	*Glehnia littoralis* Fr. Schmidtex Miq.	Glehniae Radix	Glehnia
Bihu	*Gekko swinhonis* Guenther	Gekko Swinhonis	Gekko
Binlang	*Areca catechu* L.	Arecae Semen	Areca
Buguzhi	*Psoralea corylifolia* L.	Psoraleae Fructus	Psoralea
Caodoukou	*Alpinia katsumadai* Hayata	Alpiniae Katsumadai Semen	Katsumada Galangal
Chaihu	*Bupleurum chinense* DC.	Bupleuri Radix	Bupleurum
Chenpi	*Citrus reticulata* Blanco	Citri Reticulatae Pericarpium	Tangerine Peel
Chishao	*Paeonia lactiflora* Pall.	Paeoniae Radix Rubra	Red paeony
Chuanlianzi	*Melia toosendan* Sieb.et Zucc.	Toosendan Fructus	Toosendan
Chuanxiong	*Ligusticum chuanxiong* Hort.	Chuanxiong Rhizoma	Sichuan lovase
Dahuang	*Rheum officinale* Baill.	Rhei Radix et Rhizoma	Rhei
Daxueteng	*Sargentodoxa cuneata* (Oliv.) Rehd. et Wils.	Sargentodoxae Caulis	Sargentgloryvine
Dazao	*Ziziphus jujuba* Mill.	Jujubae Fructus	Red date
Dansheng	*Salvia miltiorrhiza* Bunge	Salviae Miltiorrhizae Radix et Rhizoma	Salviae
Danggui	*Angelica sinensis* (Oliv.) Diels	Angelicae Sinensis Radix	Chinese Angelica
Dangshen	*Codonopsis pilosula* (Franch.) Nannf.	Codonopsis Radix	Codonopsis
Duhuo	*Angelica pubescens* Maxim.f. biserrata Shan et Yuan	Angelicae Pubescentis Radix	Angelicae tuhuo
Duzhong	*Eucommia ulmoides* Oliv.	Eucommiae Cortex	Eucommia
Ezhu	*Curcuma phaeocaulis* Val.	Curcumae Rhizoma	Acruginous Turmeric
Fangfeng	*Saposhnikovia divaricata* (Turcz.) Schischk.	Saposhnikoviae Radix	Divaricate Saposhniovia
Fangji	*Stephania tetrandra* S.Moore	Stephaniae Tetrandrae Radix	Fourstamen Stephania
Foshou	*Citrus medica* L. var. sarco- dactylis Swingle	Citri Sarcodactylis Fructus	Finger Citron
Fuling	*Poria cocos* (Schw.) Wolf	Poria	Poria
Fushen	*Poria cocos* (Schw.) Wolf	Poria Cum Radix Pini	Poria with hostwood
Gancao	*Glycyrrhiza uralensis* Fisch.	Glycyrrhizae Radix et Rhizoma Praeparata Cum Melle	Liquorice
Gansong	*Nardostachys jatamansi* DC.	Nardostachyos Radix et Rhizoma	Nardostachys
Ganjiang	*Zingiber officinale* Roscoe	Zingiberis Rhizoma	Dried Ginger
Gouqi	*Lycium barbarum* L.	Lycii Fructus	Wolfberry
Guya	*Setaria italica* (L.) Beauv.	Setariae Fructus Germinatus	Millet sprout
Gusuibu	*Drynaria fortunei (Kunze) J.Sm.*	Drynariae Rhizoma	Drynaria
Gualou	*Trichosanthes kirilowii* Maxim.	Trichosanthis Semen	Snakegourd seed
Guizhi	*Neolitsea cassia* (L.) Kosterm.	Cinnamomi Ramulus	Cassia twig
Hehuanpi	*Albizia julibrissin* Durazz.	Albiziae Cortex	Silktree bark
Houpu	*Magnolia officinalis* Rehder & E.H.Wilson	Magnoliae Officinalis Cortex	Magnolia bark
Huangjing	*Polygonatum kingianum* Coll.et Hemsl.	Polygonati Rhizoma	Solomonseal
Huanglian	*Coptis chinensis* Franch.	Coptidis Rhizoma	Coptis
Huangqi	*Astragalus mongholicus* Bunge	Astragali Radix	Astragalus
Huangqin	*Scutellaria baicalensis* Georgi	Scutellariae Radix	Scutellaria
Jineijin	—	Galli Gigerii Endothelium Corneum	Membrane of Chickens Gizzard
Jixueteng	*Spatholobus suberectus* Dunn	Spatholobi Caulis	Suberect Spatholobus
Jiangcan	—	Bombyx Batryticatus	Stiff Silkworm
Jiaoshenqu	—	—	Scorch-fried medicated leaven
Jinyinhua	*Lonicera japonica* Thunb.	Lonicerae Japonicae Flos	Honeysuckle
Jiegeng	*Platycodon grandiflorus* (Jacq.) A.DC.	Platycodonis Radix	Platycodon
Kushen	*Sophora flavescens* Ait.	Sophorae Flavescentis Radix	Lightyellow Sophora
Kuxingren	*Prunus armeniaca* L.	Armeniacae Semen Amarum	Bitter Apricot Seed
Lianqiao	*Forsythia suspensa* (Thunb.) Vahl	Forsythiae Fructus	Forsythia
Lianzi	*Nelumbo nucifera* Gaertn.	Nelumbinis Semen	Lotus seed
Lingzhi	*Ganoderma lucidum* (Leyss.ex Fr.) Karst.	Ganoderma	Ganoderma
Longkui	*Solanum nigrum* L.	Solani Nigri Herba	Nightshade
Maiya	*Hordeum vulgare* L.	Hordei Fructus Germinatus	Barley Sprout
Mangxiao	—	Natrii Sulfas	Mirabilite
Moyao	*Commiphora myrrha* Engl.	Myrrha	Myrrh
Muli	*Ostrea gigas* Thunberg	Ostreae Concha	Ostreae Concha
Muxiang	*Aucklandia lappa* Decne.	Aucklandiae Radix	Aucklandia
Nanshashen	*Adenophora stricta* Miq.	Adenophorae Radix	Adenophorae
Nvzhenzi	*Ligustrum lucidum* Ait.	Ligustri Lucidi Fructus	Ligustrum
Pipaye	*Eriobotrya japonica* (Thunb.) Lindl.	Eriobotryae Folium	Loquat Leaf
Pugongying	*Taraxacum mongolicum* Hand. -Mazz.	Taraxaci Herba	Dandelion
Qianghuo	*Notopterygium incisum* Ting ex H. T. Chang	Notopterygii Rhizoma et Radix	Notopterygium
Quanxie	*Buthus martensii* Karsch	Scorpio	Scorpion
Renshen	*Panax ginseng* C. A. Mey.	Ginseng Radix et Rhizoma	Ginseng
Rougui	*Cinnamomum cassia* Presl	Cinnamomi Cortex	Cassia Bark
Ruxiang	*Boswellia carterii* Birdw.	Olibanum	Frankincense
Sanleng	*Sparganium stoloniferum* Buch.-Ham.	Sparganii Rhizoma	Sparganii
Sanqi	*Panax notoginseng* (Burk.) F. H. Chen	Notoginseng Radix et Rhizoma	Sanchi
Sharen	*Amomum villosum* Lour.	Amomi Fructus	villous amomum
Shangcigu	*Cremastra appendiculata* (D.Don) Makino	Cremastrae Pseudobulbus Pleiones Pseudobulbus	Cremastra
Shandougen	*Sophora tonkinensis* Gagnep.	Sophorae Tonkinensis Radix et Rhizoma	Vietnamese Sophora Root
Shanyao	*Dioscorea opposita* Thunb.	Dioscoreae Rhizoma	Common Yam
Shanzha	*Crataegus pinnatifida* Bge.	Crataegi Fructus	Crataegi
Shanzhuyu	*Cornus officinalis* Sieb. et Zucc.	Corni Fructus	Cornus
Shengma	*Cimicifuga heracleifolia* Kom.	Cimicifugae Rhizoma	Cimicifuga
Shengjiang	*Zingiber officinale* Roscoe	Zingiberis Rhizoma Recens	Raw Ginger
Shidachuan	*Hedyotis chrysotricha* (Palib.) Merr.	Hedyotis Chrysotrichae Herba	Hedyotis Chrysotricha
Shihu	*Dendrobium nobile* Lindl.	Dendrobii Caulis	Dendrobium
Shudihuang	*Rehmannia glutinosa* Libosch.	Rehmanniae Radix Praeparata	Rehmannia Glutinosa
Suanzaoren	*Ziziphus jujuba* Mill. var. spinosa (Bunge) Hu ex H. F. Chou	Ziziphi Spinosae Semen	Spine Date Seed
Taizishen	*Pseudostellaria heterophylla* (Miq.) Pax	Pseudostellariae Radix	Pseudostellaria
Taoren	*Prunus persica* (L.) Batsch	Persicae Semen	Peach kernel
Tengligen	*Actinidia chinensis* Planch. var. hispida C.F.Liang	Actinidiae Chinensis Radix	Actinidia root
Tusizi	*Cuscuta chinensis* Lam.	Cuscutae Semen	Cuscuta
Walengzi	*Arca subcrenata* Lischke	Arcae Concha	Arcae concha
Weilingxian	*Clematis chinensis* Osbeck	Clematidis Radix et Rhizoma	Chinese Clematis
Wumei	*Prunus mume* (Siebold) Siebold et Zucc.	Mume Fructus	Dark plum
Wugong	*Scolopendra subspinipes* mutilans L. Koch	Scolopendra	Scolopendra
Xiakucao	*Prunella vulgaris* L.	Prunellae Spica	Prunella
Xianmao	*Curculigo orchioides* Gaertn.	Curculiginis Rhizoma	Curculigo
Xuanshen	*Scrophularia ningpoensis* Hemsl.	Scrophulariae Radix	Figwort
Xuanfuhua	*Inula japonica* Thunb.	Inulae Flos	Inula
Yeputaoteng	*Vitis amurensis* Rupr.	Vitis Amurensis Caulis	Amur grape vines
Yiyiren	*Coix lacryma-jobi* L.	Coicis Semen	Coxi seed
Yinyanghuo	*Epimedium brevicornu* Maxim.	Epimedii Folium	Epimedium
Yanhusuo	*Corydalis yanhusuo* W.T.Wang	Corydalis Rhizoma	Corydalis
Zaojiaoci	*Gleditsia sinensis* Lam.	Gleditsiae Spina	Gleditsia sinensis
Zexie	*Alisma plantago-aquatica* subsp. orientale (Sam.) Sam.	Alismatis Rhizoma	Alisma orientale
Zhebeimu	*Fritillaria thunbergii* Miq.	Fritillariae Thunbergii Bulbus	Thunberbg fritillary
Zhiqiao	*Citrus × aurantium* L.	Aurantii Fructus	Aurantii Fructus
Zhishi	*Citrus × aurantium* L.	Fructus Aurantii Immaturus	Aurantii Fructus Immaturus
Chonglou	*Paris polyphylla* Smith var.yunnanensis(Franch.)Hand.-Mazz.	Paridis Rhizoma	Paris Polyphylla
Zhuling	*Polyporus umbellatus* (Pers.) Fries	Polyporus	Agaric
Zhuru	*Bambusa tuldoides* Munro	Bambusae Caulis in Taenias	Bambusae Caulis
Zisugeng	*Perilla frutescens* (L.) Britt.	Perillae Caulis	Perilla frutescens stem

#### 3.1.2 Risk of bias of included trials

**FIGURE 2:**
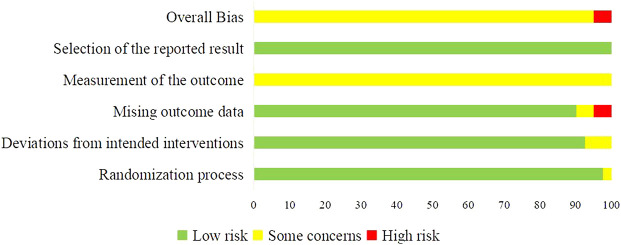
Summary of risk of bias of included trials.

RoB 2 was used to assess the quality of 40 included trials. Two trials were assessed as “High” risk of bias (Zhao, 2011; Zhang, 2019), and 38 trials were assessed as “Some concerns” (Chen et al., 2007; Chi et al., 2010; Hu, 2011; Yuan, 2011; Zhu et al., 2011; Qin, 2012; Guo, 2014; Huang, 2014; Liu et al., 2015; Li et al., 2016; Zhao et al., 2016; Chu et al., 2017; Feng, 2017; Huang et al., 2017; Wang Y. et al., 2018; Yang et al., 2018; Yuan, 2018; Cai et al., 2019; Gu et al., 2019; Jiao, 2019; Liu et al., 2019; Xie, 2019; Yu et al., 2019; Zhai, 2019; Zhong et al., 2019; Bao, 2020; Gong, 2020; Li D. H. et al., 2020; Sun et al., 2020; Zhang et al., 2020; Zhang, 2021a; Zhang, 2021b; Feng et al., 2021; Jiang et al., 2021; Long, 2021; Zhang L. et al., 2021; Zhao, 2021; Zhong et al., 2021). Most concerns were caused by the measurement of the outcomes, since the assessment of outcomes could be influenced by knowledge of interventions patients received. The imbalanced missing data in two trials lead to the “High” risk in domain of missing outcome data, and in overall bias. The summary of RoB was shown in Figure 2.

### 3.2 The tumor response of CHM combined with oxaliplatin-based chemotherapy in AGC

**FIGURE 3:**
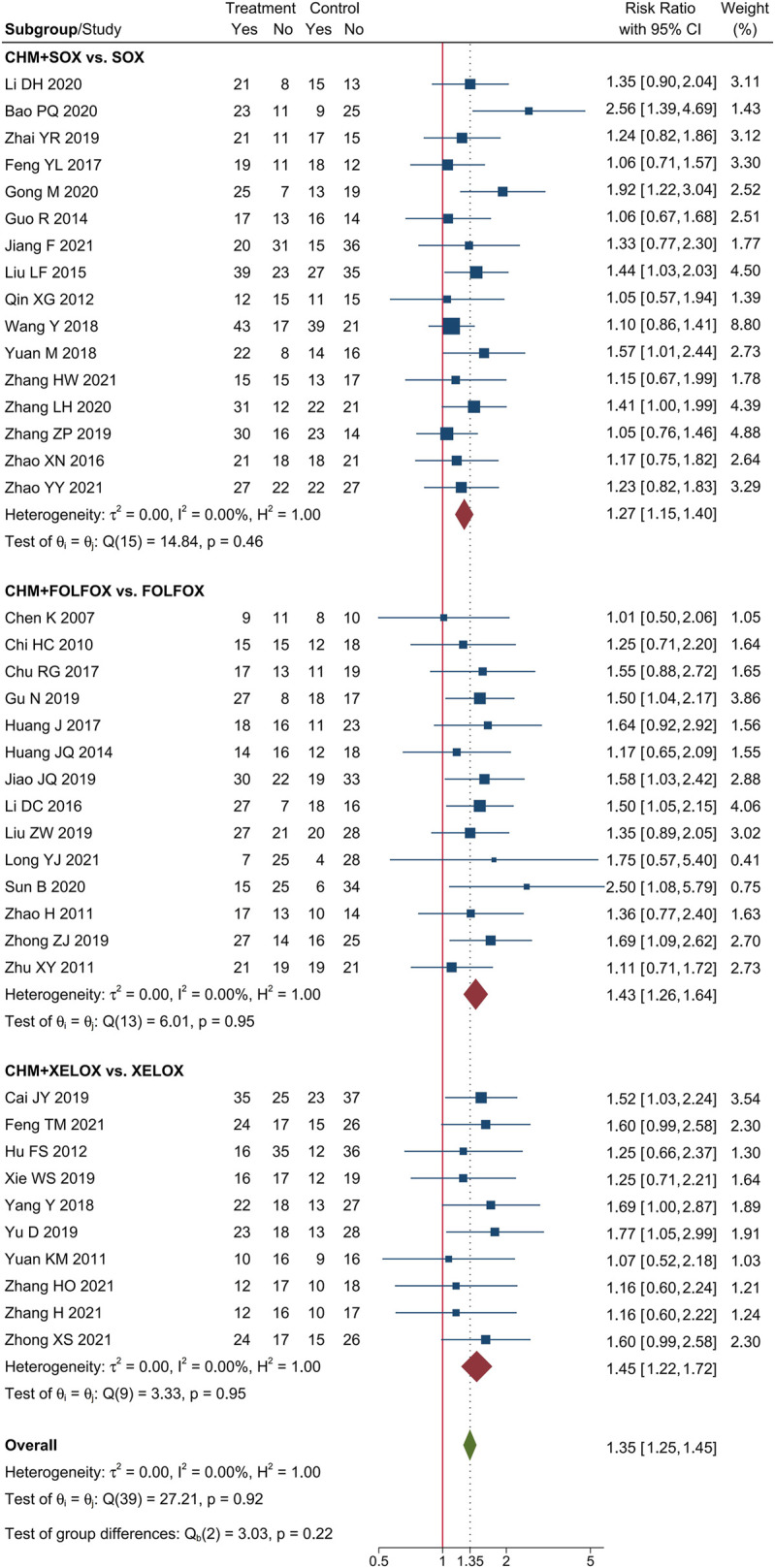
Forest plot of ORR.

**FIGURE 4:**
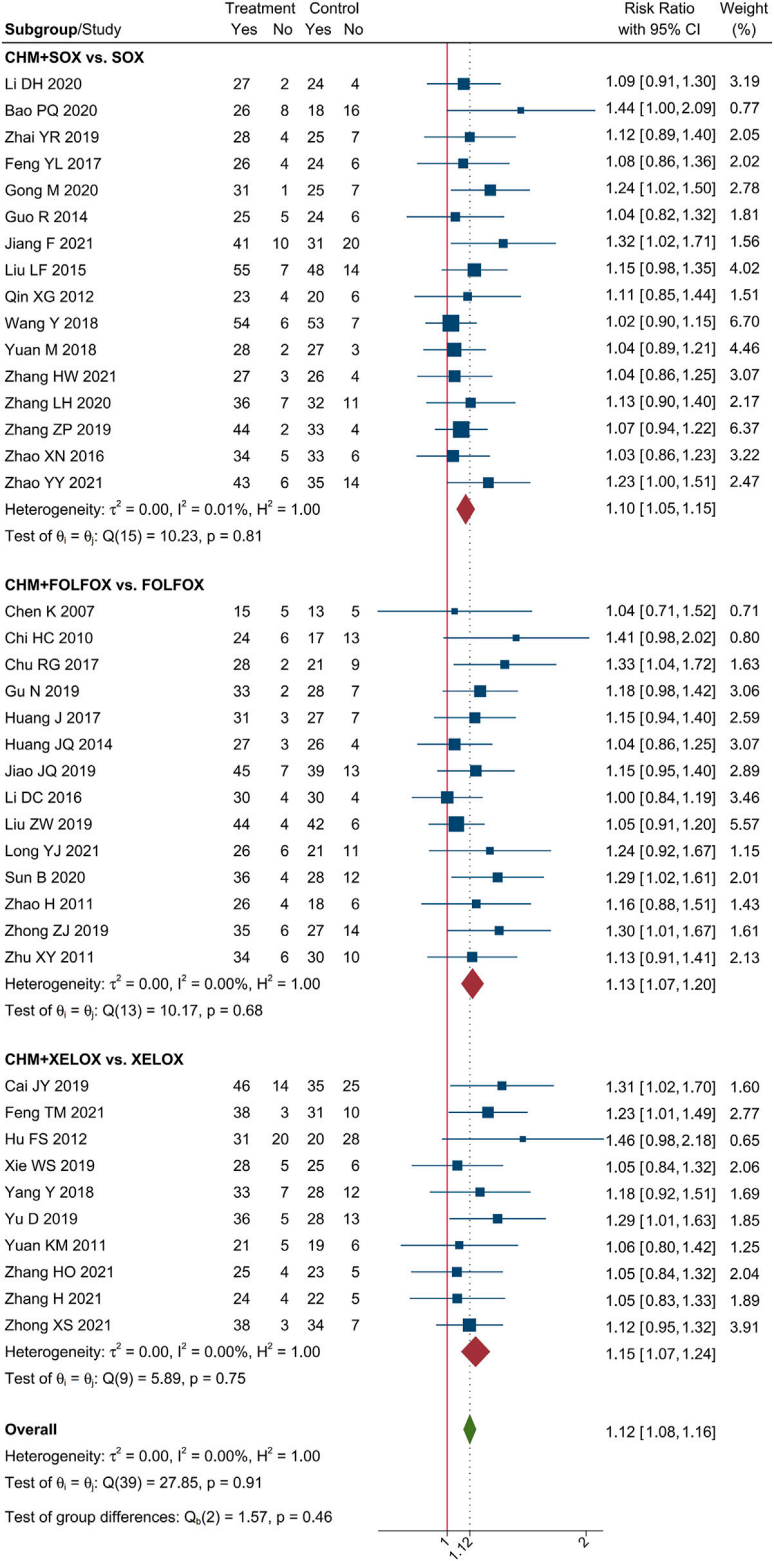
Forest plot of DCR.

Meta-analysis of 40 trials showed that CHM combined with oxaliplatin-based chemotherapy could increase the ORR by 35% [RR = 1.35, 95% CI (1.25, 1.45)]. The value of I^2^ = 0 indicates that there was no statistical heterogeneity among these trials. The forest plot of ORR was shown in Figure 3. Meta-analysis of 40 trials showed that CHM combined with oxaliplatin-based chemotherapy could increase the DCR by 12% [RR = 1.12, 95% CI (1.08, 1.16)]. I^2^ = 0 indicates that there was no statistical heterogeneity among these trials. The forest plot of DCR was shown in Figure 4.

### 3.3 The safety of CHM combined with oxaliplatin-based chemotherapy in AGC

Safety outcomes were reported in 37 trials. We evaluated the incidence of AEs of blood system, gastrointestinal reaction, hepatorenal toxicity, and peripheral neurotoxicity.

#### 3.3.1 AEs of blood system

**FIGURE 5:**
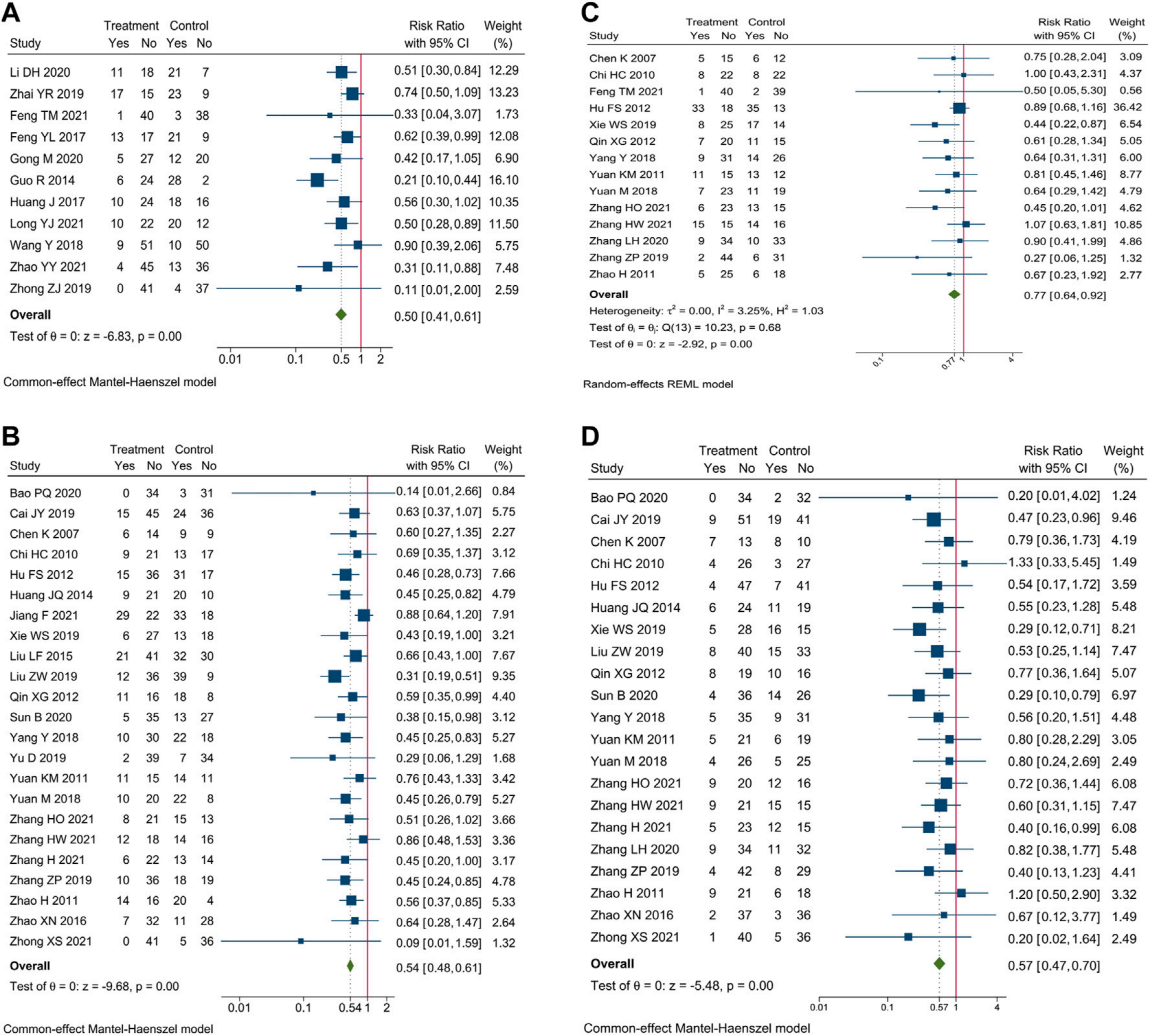
Forest plot of incidence of AEs of blood system. Incidence of **(A)** myelosuppression, **(B)** leucopenia, **(C)** anemia, and **(D)** thrombocytopenia.

Incidence of AEs of blood system were reported in 35 trials. Meta-analysis showed that compare oxaliplatin-based chemotherapy alone, CHM combined with oxaliplatin-based chemotherapy could decrease the incidence of myelosuppression by 50% [RR = 0.50, 95% CI (0.41, 0.61)], leucopenia by 46% [RR = 0.54, 95% CI (0.48, 0.61)], anemia by 23% [RR = 0.77, 95% CI (0.64, 0.92)], and thrombocytopenia by 43% [RR = 0.57, 95% CI (0.47, 0.70)]. Forest plots of incidence of AEs of blood system were shown in Figure 5.

#### 3.3.2 Gastrointestinal reaction

**FIGURE 6:**
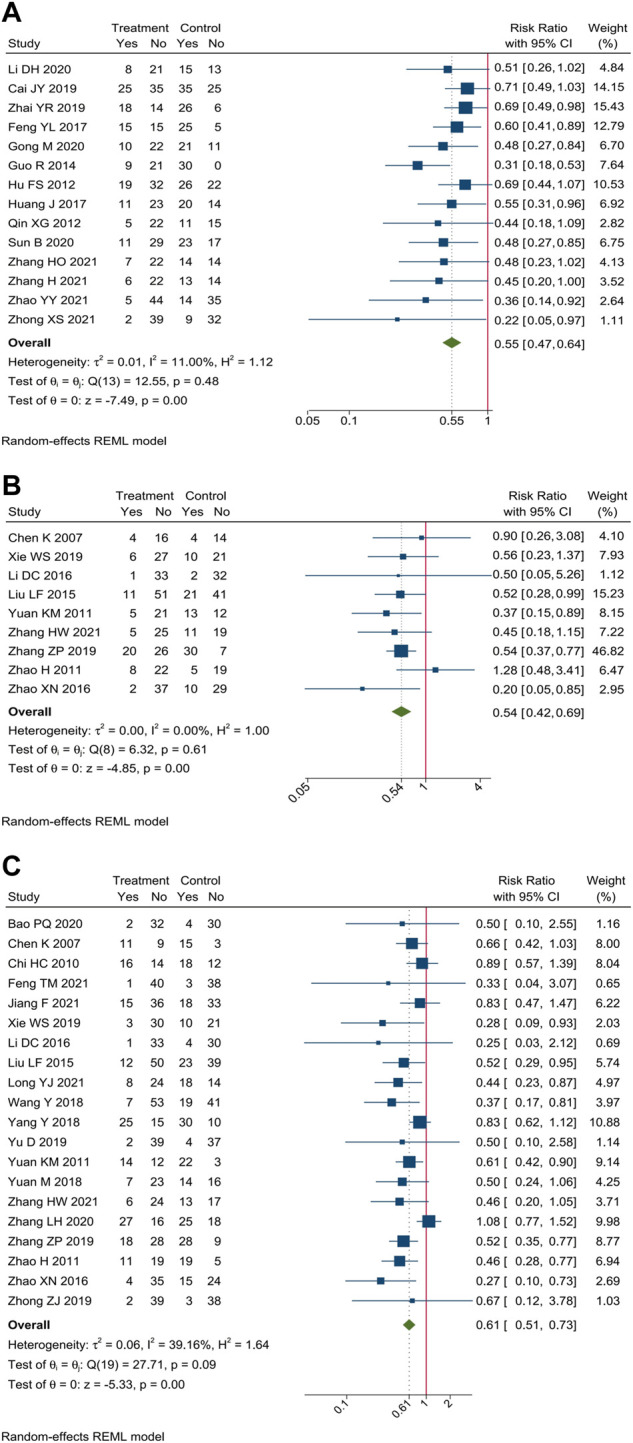
Forest plot of incidence of gastrointestinal reaction. Incidence of **(A)** gastrointestinal reaction, **(B)** diarrhea, and **(C)** nausea and vomiting.

Incidence of gastrointestinal reaction were reported in 34 trials. Meta-analysis showed that compare oxaliplatin-based chemotherapy alone, CHM combined with oxaliplatin-based chemotherapy could decrease the incidence of gastrointestinal reaction by 45% [RR = 0.55, 95% CI (0.47, 0.64)], nausea and vomiting by 39% [RR = 0.61, 95% CI (0.51, 0.73)], and diarrhea by 46% [RR = 0.54, 95% CI (0.42, 0.69)]. Forest plots of incidence of gastrointestinal reaction were shown in Figure 6.

#### 3.3.3 Hepatorenal toxicity

**FIGURE 7:**
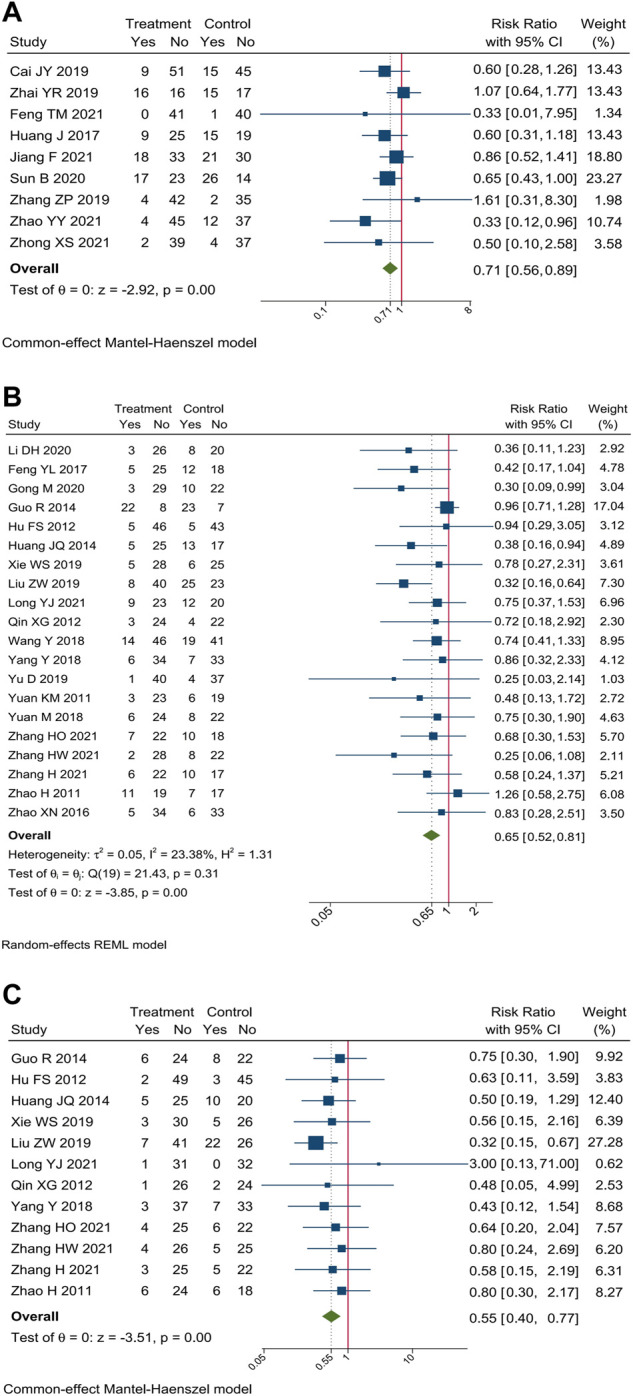
Forest plot of incidence of hepatorenal toxicity. Incidence of **(A)** hepatorenal toxicity, **(B)** hepatotoxicity, and **(C)** renal toxicity.

Incidence of hepatorenal toxicity were reported in 29 trials. Meta-analysis showed that compare oxaliplatin-based chemotherapy alone, CHM combined with oxaliplatin-based chemotherapy could decrease the incidence of hepatorenal toxicity by 29% [RR = 0.71, 95% CI (0.56, 0.89)], hepatotoxicity by 35% [RR = 0.65, 95% CI (0.52, 0.81)], and renal toxicity by 45% [RR = 0.55, 95% CI (0.40, 0.77)]. Forest plots of incidence of hepatorenal toxicity were shown in Figure 7.

#### 3.3.4 Peripheral neurotoxicity

**FIGURE 8:**
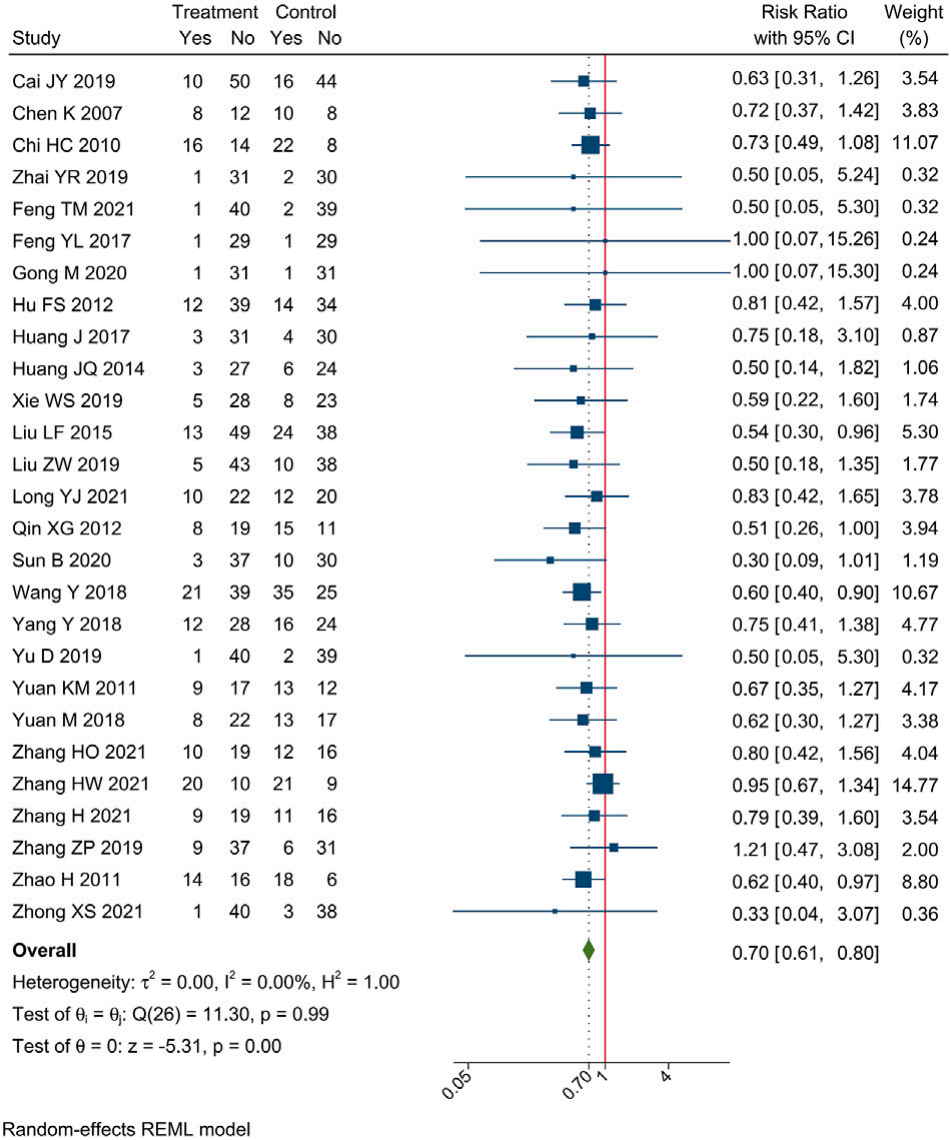
Forest plot of incidence of peripheral neurotoxicity.

Incidences of peripheral neurotoxicity were reported in 27 trials. Meta-analysis showed that compare oxaliplatin-based chemotherapy alone, CHM combined with oxaliplatin-based chemotherapy could decrease the incidence of peripheral neurotoxicity by 30% [RR = 0.70, 95% CI (0.61, 0.80)]. Forest plots of incidence of hepatorenal toxicity were shown in Figure 8.

### 3.4 Subgroup analysis

We performed subgroup analysis of the outcomes of tumor-response, according to the different regimen of chemotherapy that patients received. Compare to SOX regimen alone, CHM combined with SOX regimen chemotherapy could increase the ORR by 27% [RR = 1.27, 95% CI (1.15, 1.40)], and DCR by 10% [RR = 1.10, 95% CI (1.05, 1.15)]. Compare to FOLFOX regimen alone, CHM combined with FOLFOX regimen chemotherapy could increase the ORR by 43% [RR = 1.43, 95% CI (1.26, 1.64)], and DCR by 14% [RR = 1.13, 95% CI (1.07, 1.20)]. Compare to XELOX regimen alone, CHM combined with XELOX regimen chemotherapy could increase the ORR by 27% [RR = 1.35, 95% CI (1.26, 1.45)], and DCR by 12% [RR = 1.12, 95% CI (1.09, 1.16)]. The details were shown in Figures 3, 4.

### 3.5 Sensitivity analysis

We performed sensitivity analysis to investigate the potential contributions of specific herbs to tumor response. Three trials which adopted multiple core prescriptions were excluded from sensitivity analysis, and we analyzed the composition of core prescriptions in other 37 trials that adopted single core prescription. There are 114 herbs in the formulas of included trials, 47 of these herbs were used only in one trial and were excluded from the sensitivity analysis. Among 67 herbs analyzed, 43 herbs were only used with other herbs as a combination, which means that when herb was used in several formulas, there was always an herb in the same formulas. RRs of the group of trials that included each specific herb or herb combination were calculated. Among 380 sensitivity analyses performed, 21 single herbs and 129 herb combinations were located, and 3 single herbs along with 227 herb combinations were excluded according to the predetermined principle.

#### 3.5.1 Tier 1: Single herbs

The five most frequently used single herbs were Largehead Atractylodes (*n* = 26), Liquorice (*n* = 23), Poria (*n* = 21), Pinellia (*n* = 20), and Codonopsis Pilosula (*n* = 18). The single herbs with the five highest RR were Dandelion [RR = 1.47, 95% CI (1.17, 1.83), *n* = 5], Paris Polyphylla [RR = 1.47, 95% CI (1.07, 2.02), *n* = 3], Red Paeony [RR = 1.46, 95% CI (1.13, 1.88) *n* = 4], Chinese Angelica [RR = 1.44, 95% CI (1.27, 1.64), *n* = 14], and Astragalus [RR = 1.43, 95% CI (1.27, 1.64), *n* = 13]. Other details of the contributions of single herbs were shown in Table 4.

**TABLE 4:** Sensitivity analysis of specific contributions of herbs.

Tier	Composition	No. of RCTs	Effect estimates (EE)	95% CI of EE	I^2^ statistics (%)
1	Dandelion	5	1.47	1.17, 1.83	0.00
1	Paris Polyphylla	3	1.47	1.07, 2.02	10.95
1	Red paeony	4	1.46	1.13, 1.88	0.00
1	Chinese Angelica	14	1.44	1.27, 1.64	9.33
1	Astragalus	13	1.43	1.25, 1.64	0.00
1	Scutellaria Barbata	8	1.41	1.18, 1.69	28.11
1	Villous Amomum	7	1.40	1.13, 1.73	33.15
1	Smilax	6	1.40	1.17, 1.67	8.17
1	White Paeony	10	1.39	1.20, 1.61	14.62
1	Ginseng	6	1.39	1.17, 1.65	0.00
1	Codonopsis	18	1.38	1.23, 1.55	5.92
1	Acruginous Turmeric	15	1.36	1.20, 1.54	16.12
1	Poria	21	1.35	1.23,1.49	0.00
1	Largehead Atractylodes	26	1.34	1.23,1.47	0.00
1	Gekko	6	1.33	1.10, 1.60	14.72
1	Tangerine Peel	17	1.32	1.18, 1.47	6.29
1	Hedyotis Diffusa	12	1.32	1.16, 1.50	3.56
1	Membrane of Chickens Gizzard	7	1.32	1.11, 1.57	0.00
1	Salviae	4	1.32	1.04, 1.67	0.00
1	Liquorice	23	1.30	1.18, 1.42	0.00
1	Pinellia	20	1.28	1.17, 1.41	0.00
2	Codonopsis + Villous Amomum	4	1.70	1.34, 2.16	0.00
2	Ginseng + Cornus	2	1.56	1.16, 2.10	0.00
2	Acruginous Turmeric + Astragalus	7	1.55	1.28, 1.88	0.00
2	Chinese Angelica + Astragalus	7	1.55	1.28, 1.89	0.00
2	Chinese Angelica + Smilax	5	1.55	1.27, 1.88	0.00
2	Acruginous Turmeric + Cassia Twig	4	1.54	1.23, 1.91	0.00
2	Scutellaria Barbata + Membrane of Chickens Gizzard	3	1.53	1.18, 1.98	0.00
2	Chinese Angelica + Myrrh	3	1.52	1.15, 2.00	0.00
2	Largehead Atractylodes + Wolfberry	4	1.51	1.22, 1.89	0.00
2	Astragalus + Villous Amomum	4	1.50	1.11, 2.03	25.93
2	Codonopsis + Astragalus	8	1.47	1.23, 1.76	0.00
2	Liquorice + Bupleurum	5	1.46	1.19, 1.79	0.00
2	Codonopsis + Chinese Angelica	8	1.46	1.20, 1.77	27.05
2	Codonopsis + Aucklandia	6	1.46	1.20, 1.77	0.00
2	Largehead Atractylodes + Scutellaria Barbata	7	1.44	1.19, 1.74	27.24
2	Largehead Atractylodes + White Paeony	9	1.41	1.21, 1.63	12.42
2	Largehead Atractylodes + Ginseng	3	1.41	1.21, 1.78	0.00
2	Largehead Atractylodes + Membrane of Chickens Gizzard	5	1.40	1.14, 1.73	0.00
2	Acruginous Turmeric + Hedyotis Diffusa	8	1.40	1.16, 1.70	21.43
2	Membrane of Chickens Gizzard + Gekko	4	1.40	1.12, 1.76	0.00
2	Largehead Atractylodes + Yam	9	1.38	1.18, 1.62	13.56
2	Liquorice + Poria	17	1.36	1.22, 1.52	0.00
2	Pinellia + Gekko	5	1.35	1.10, 1.65	14.01
2	Pinellia + Magnolia Bark	4	1.30	1.04, 1.64	0.00
2	Pinellia + Dried Ginger	4	1.29	1.03, 1.61	1.47
3	Chinese Angelica + Astragalus + Villous Amomum	3	1.73	1.26, 2.36	0.00
3	Largehead Atractylodes + Wolfberry + Psoralea	2	1.65	1.24, 2.19	0.00
3	Codonopsis + Chinese Angelica + Astragalus	6	1.60	1.30, 1.97	0.00
3	Liquorice + Codonopsis + Bupleurum	3	1.57	1.14, 2.15	0.00
3	Pinellia + White Paeony + Dried Ginger	2	1.56	1.10, 2.21	0.00
3	Codonopsis + Acruginous Turmeric + Astragalus	5	1.53	1.22, 1.91	0.00
3	Largehead Atractylodes + Astragalus + White Paeony	5	1.52	1.26, 1.84	0.00
3	Largehead Atractylodes + Poria + Aucklandia	5	1.51	1.22, 1.86	0.00
3	Largehead Atractylodes + Acruginous Turmeric + Membrane of Chickens Gizzard	4	1.51	1.19, 1.91	0.00
3	Acruginous Turmeric + Chinese Angelica + Astragalus	5	1.51	1.21, 1.89	0.00
3	Acruginous Turmeric + Hedyotis Diffusa + Red paeony	3	1.51	1.14, 2.01	0.00
3	Scutellaria Barbata + Membrane of Chickens Gizzard + Gekko	2	1.51	1.11, 2.04	0.00
3	Largehead Atractylodes + Pinellia + Scutellaria Barbata	5	1.50	1.18, 1.92	39.55
3	Largehead Atractylodes + Astragalus + Villous Amomum	2	1.50	1.04, 2.16	0.00
3	Largehead Atractylodes + Pinellia + Dandelion	4	1.49	1.17, 1.91	0.00
3	Largehead Atractylodes + Acruginous Turmeric + Astragalus	6	1.47	1.20, 1.80	0.00
3	Liquorice + Codonopsis + Aucklandia	5	1.47	1.15, 1.86	0.00
3	Largehead Atractylodes + Acruginous Turmeric + Scutellaria Barbata	4	1.46	1.12, 1.89	39.92
3	Largehead Atractylodes + Chinese Angelica + Astragalus	6	1.46	1.19, 1.80	0.00
3	Largehead Atractylodes + Astragalus + Coxi Seed	6	1.46	1.21, 1.78	0.00
3	Largehead Atractylodes + Pinellia + Astragalus	5	1.44	1.17, 1.77	0.00
3	Largehead Atractylodes + Tangerine Peel + White Paeony	6	1.44	1.19, 1.74	27.78
3	Acruginous Turmeric + Hedyotis Diffusa + Cassia Twig	3	1.44	1.12, 1.84	0.00
3	Largehead Atractylodes + Pinellia + White Paeony	6	1.43	1.15, 1.77	29.02
3	Codonopsis + White Paeony + Aucklandia	3	1.41	1.09, 1.82	0.00
3	Liquorice + White Paeony + Bupleurum	4	1.40	1.11, 1.76	0.00
3	Largehead Atractylodes + Poria + Suberect Spatholobus	3	1.39	1.05, 1.83	0.00
3	Pinellia + Tangerine Peel + Barley Sprout	5	1.39	1.10, 1.75	4.55
3	Pinellia + Gekko + Magnolia Bark	3	1.39	1.06, 1.81	0.00
3	Largehead Atractylodes + Tangerine Peel + Yam	6	1.38	1.13, 1.69	22.74
3	Largehead Atractylodes + Liquorice + Poria	16	1.37	1.22, 1.53	0.00
3	Largehead Atractylodes + Pinellia + Membrane of Chickens Gizzard	4	1.36	1.08, 1.72	0.00
4	Codonopsis + Chinese Angelica + Astragalus + Villous Amomum	2	1.95	1.36, 2.78	0.00
4	Largehead Atractylodes + Pinellia + Tangerine Peel + Dandelion	2	1.76	1.24, 2.52	0.00
4	Acruginous Turmeric + Chinese Angelica + Astragalus + Villous Amomum	2	1.75	1.01, 3.03	34.11
4	Liquorice + Pinellia + Tangerine Peel + Paris Polyphylla	2	1.74	1.18, 2.56	0.00
4	Largehead Atractylodes + Tangerine Peel + Astragalus + Yam	4	1.63	1.30, 2.05	0.00
4	Codonopsis + Acruginous Turmeric + Chinese Angelica + Astragalus	4	1.57	1.24, 1.99	0.00
4	Largehead Atractylodes + Liquorice + Poria + Aucklandia	4	1.55	1.19, 2.02	0.00
4	Largehead Atractylodes + Tangerine Peel + Acruginous Turmeric + Astragalus	4	1.54	1.23, 1.93	0.00
4	Largehead Atractylodes + Poria + Chinese Angelica + Astragalus	5	1.53	1.23, 1.91	0.00
4	Largehead Atractylodes + Astragalus + Wolfberry + Cuscuta	3	1.53	1.12, 2.08	0.00
4	Acruginous Turmeric + Chinese Angelica + Sparganii + Smilax	4	1.53	1.24, 1.89	0.00
4	Largehead Atractylodes + Liquorice + Poria + Villous Amomum	4	1.52	1.19, 1.93	0.00
4	Largehead Atractylodes + Liquorice + Poria + Bupleurum	4	1.51	1.22, 1.89	0.00
4	Largehead Atractylodes + Poria + Codonopsis + Aucklandia	5	1.51	1.22, 1.86	0.00
4	Largehead Atractylodes + Pinellia + Tangerine Peel + Barley Sprout	4	1.50	1.15, 1.97	3.91
4	Largehead Atractylodes + Codonopsis + Chinese Angelica + Astragalus	5	1.50	1.21, 1.87	0.00
4	Largehead Atractylodes + Liquorice + Chinese Angelica + Astragalus	5	1.48	1.14, 1.02	0.00
4	Largehead Atractylodes + Pinellia + Tangerine Peel + Astragalus	4	1.47	1.19, 1.83	0.00
4	Liquorice + Pinellia + Tangerine Peel + Ginseng	2	1.47	1.12, 1.93	0.00
4	Largehead Atractylodes + Poria + Tangerine Peel + Astragalus	6	1.45	1.21, 1.73	0.00
4	Largehead Atractylodes + Poria + Codonopsis + Astragalus	6	1.44	1.18, 1.75	0.00
4	Liquorice + Pinellia + Codonopsis + Aucklandia	3	1.42	1.08, 1.85	0.00
4	Liquorice + Pinellia + White Paeony + Bupleurum	3	1.42	1.08, 1.88	0.00
4	Largehead Atractylodes + Poria + Codonopsis + White Paeony	5	1.40	1.13, 1.73	23.16
4	Largehead Atractylodes + Acruginous Turmeric + Coxi Seed + Yam	4	1.39	1.12, 1.72	30.94
4	Largehead Atractylodes + Liquorice + Poria + Aurantii Fructus	3	1.38	1.05, 1.81	0.00
4	Largehead Atractylodes + Acruginous Turmeric + Membrane of Chickens Gizzard + Crataegi	3	1.38	1.05, 1.82	0.00
5	Largehead Atractylodes + Pinellia + Acruginous Turmeric + Astragalus + White Paeony	3	1.67	1.23, 2.27	0.00
5	Largehead Atractylodes + Acruginous Turmeric + Astragalus + Wolfberry + Cuscuta	2	1.66	1.15, 2.41	0.00
5	Largehead Atractylodes + Liquorice + Poria + Chinese Angelica + Astragalus	4	1.59	1.19, 2.13	0.00
5	Acruginous Turmeric + Chinese Angelica + Hedyotis Diffusa + Sparganii + Smilax	3	1.59	1.22, 2.08	0.00
5	Largehead Atractylodes + Liquorice + Poria + Codonopsis + Villous Amomum	3	1.58	1.22, 2.05	0.00
5	Largehead Atractylodes + Liquorice + Poria + Tangerine Peel + Bupleurum	3	1.58	1.22, 2.05	0.00
5	Largehead Atractylodes + Liquorice + Poria + Codonopsis + Aucklandia	4	1.55	1.19, 2.02	0.00
5	Largehead Atractylodes + Codonopsis + Tangerine Peel + Chinese Angelica + Astragalus	5	1.50	1.21, 1.87	0.00
5	Largehead Atractylodes + Poria + Codonopsis + Tangerine Peel + Aucklandia	3	1.49	1.12, 1.97	0.00
5	Largehead Atractylodes + Poria + Codonopsis + Acruginous Turmeric + Astragalus	3	1.48	1.14, 1.93	0.00
5	Largehead Atractylodes + Liquorice + Poria + White Paeony + Bupleurum	3	1.46	1.13, 1.88	0.00
5	Largehead Atractylodes + Liquorice + Poria + Pinellia + Coptis	2	1.45	1.08, 1.94	0.00
5	Largehead Atractylodes + Liquorice + Poria + Tangerine Peel + Astragalus	5	1.45	1.17, 1.78	0.00
5	Largehead Atractylodes + Liquorice + Poria + Codonopsis + White Paeony	4	1.44	1.07, 1.93	33.23
5	Largehead Atractylodes + Poria + Codonopsis + Hedyotis Diffusa + Suberect Spatholobus	2	1.43	1.04, 1.97	0.00
5	Largehead Atractylodes + Liquorice + Poria + Hedyotis Diffusa + Scutellaria Barbata	5	1.42	1.14, 1.77	26.53
6	Largehead Atractylodes + Pinellia + Tangerine Peel + White Paeony + Scutellaria Barbata + Barley Sprout	2	2.04	1.37, 3.05	0.00
6	Largehead Atractylodes + Liquorice + Poria + Codonopsis + Tangerine Peel + Bupleurum	2	1.84	1.24, 2.72	0.00
6	Largehead Atractylodes + Acruginous Turmeric + Coxi Seed + Yam + Scutellaria Barbata + Membrane of Chickens Gizzard	2	1.76	1.27, 2.45	0.00
6	Largehead Atractylodes + Pinellia + Coxi Seed + Inula + Haematitum	2	1.68	1.25, 2.25	0.00
6	Largehead Atractylodes + Poria + Codonopsis + Tangerine Peel + Chinese Angelica + Astragalus	4	1.59	1.25, 2.01	0.00
6	Largehead Atractylodes + Liquorice + Codonopsis + Tangerine Peel + Chinese Angelica + Astragalus	4	1.55	1.16, 2.06	0.00
6	Largehead Atractylodes + Liquorice + Poria + Pinellia + Codonopsis + Villous Amomum	2	1.53	1.11, 2.10	0.00
6	Largehead Atractylodes + Liquorice + Poria + White Paeony + Bupleurum + Aurantii Fructus	2	1.52	1.05, 2.20	0.00
6	Largehead Atractylodes + Poria + Chinese Angelica + Coxi Seed + Yam + Smilax	3	1.52	1.18, 1.95	0.00
6	Largehead Atractylodes + Liquorice + Poria + Pinellia + Codonopsis + Aucklandia	2	1.51	1.11, 2.06	0.00
6	Largehead Atractylodes + Liquorice + Poria + Tangerine Peel + Astragalus + Bupleurum	2	1.51	1.15, 1.98	0.00
6	Largehead Atractylodes + Poria + Codonopsis + Chinese Angelica + Yam + Aucklandia	3	1.51	1.13, 2.00	0.00
6	Largehead Atractylodes + Tangerine Peel + Acruginous Turmeric + Barley Sprout + Cassia Twig + Dried Ginger	2	1.49	1.10, 2.03	3.12
6	Largehead Atractylodes + Pinellia + Acruginous Turmeric + Membrane of Chickens Gizzard + Gekko + Dandelion	3	1.48	1.13, 1.95	0.00
6	Largehead Atractylodes + Liquorice + Poria + Codonopsis + Tangerine Peel + Astragalus	4	1.47	1.13, 1.91	0.00
6	Largehead Atractylodes + Poria + Acruginous Turmeric + Chinese Angelica + Astragalus + White Paeony	3	1.47	1.13, 1.91	0.00
6	Largehead Atractylodes + Liquorice + Poria + Pinellia + Tangerine Peel + Coptis	2	1.45	1.08, 1.94	0.00
7	Largehead Atractylodes + Liquorice + Poria + Codonopsis + Tangerine Peel + Chinese Angelica + Astragalus	3	1.75	1.25, 2.38	0.00
7	Largehead Atractylodes + Liquorice + Poria + Pinellia + Tangerine Peel + White Paeony + Bupleurum	2	1.53	1.11, 2.11	0.00
7	Largehead Atractylodes + Liquorice + Poria + Chinese Angelica + Coxi Seed + Yam + Smilax	2	1.61	1.12, 2.34	0.00
7	Largehead Atractylodes + Pinellia + Tangerine Peel + Acruginous Turmeric + Astragalus + White Paeony + Scutellaria Barbata	2	1.86	1.31, 2.62	0.00
8	Largehead Atractylodes + Liquorice + Poria + Pinellia + Tangerine Peel + Astragalus + White Paeony + Alisma Orientale	2	1.51	1.13, 2.02	0.00
8	Largehead Atractylodes + Poria + Codonopsis + Tangerine Peel + Acruginous Turmeric + Chinese Angelica + Astragalus + White Paeony	2	1.53	1.15, 2.04	0.00
8	Largehead Atractylodes + Poria + Codonopsis + Tangerine Peel + Chinese Angelica + Astragalus + Yam + Aucklandia	2	1.47	1.06, 2.04	0.00
8	Largehead Atractylodes + Poria + Acruginous Turmeric + Chinese Angelica + Coxi Seed + Yam + Sparganii + Smilax	2	1.50	1.13, 1.97	0.00
9	Largehead Atractylodes + Liquorice + Poria + Codonopsis + Tangerine Peel + Chinese Angelica + Astragalus + Yam + Lotus Seed	2	1.70	1.12, 2.56	0.00
9	Acruginous Turmeric + Chinese Angelica + Hedyotis Diffusa + Sparganii + Smilax + Red paeony + Cassia Twig + Myrrh + Frankincense	2	1.59	1.15, 2.18	0.00
10	Largehead Atractylodes + Poria + Codonopsis + Chinese Angelica + Coxi Seed + White Paeony + Yam + Aucklandia + Smilax + Hedyotis Chrysotricha	2	1.49	1.11, 2.00	0.00

#### 3.5.2 Tier 2: Combination of 2 herbs

The three most frequently used combination of 2 herbs were: Liquorice + Poria (*n* = 17), Largehead Atractylodes + White Paeony (*n* = 9), and Largehead Atractylodes + Yam (*n* = 9). The combination of 2 herbs with the five highest RR were Codonopsis Pilosula + Villous Amomum [RR = 1.70, 95% CI (1.34, 2.16), *n* = 4], Cornus + Ginseng [RR = 1.56, 95% CI (1.16,2.10), *n* = 2], Acruginous Turmeric + Astragalus [RR = 1.55, 95% CI (1.28, 1.88), *n* = 7], Chinese Angelica + Astragalus [RR = 1.55, 95% CI (1.28, 1.89), *n* = 7], and Chinese Angelica + Smilax [RR = 1.55, 95% CI (1.27, 1.88), *n* = 5]. Other sensitivity analysis of combination of 2 herbs were shown in Table 4.

#### 3.5.3 Tier 3: Combination of 3 herbs

The combination of 3 herbs with the five highest RR were Chinese Angelica + Astragalus + Villous Amomum [RR = 1.73, 95% CI (1.26, 2.36), *n* = 3], Largehead Atractylodes + Wolfberry + Psoralea [RR = 1.65, 95% CI (1.24, 2.19), *n* = 2], Codonopsis + Chinese Angelica + Astragalus [RR = 1.60, 95% CI (1.30, 1.97), *n* = 6], Liquorice + Codonopsis + Bupleurum [RR = 1.57, 95% CI (1.14, 2.15), *n* = 3], and Pinellia + White Paeony + Dried Ginger [RR = 1.56, 95% CI (1.10, 2.21), *n* = 2]. Other sensitivity analysis of combination of 3 herbs were shown in Table 4.

#### 3.5.4 Tier 4: Combination of 4 herbs

Two combination of 4 herbs were used more than five times: Largehead Atractylodes + Poria + Tangerine Peel + Astragalus (*n* = 6), and Largehead Atractylodes + Poria + Codonopsis + Astragalus (*n* = 6). The combination of 3 herbs with the five highest RR were Codonopsis + Chinese Angelica + Astragalus + Villous Amomum [RR = 1.95, 95% CI (1.36, 2.78), n = 2], Largehead Atractylodes + Pinellia + Tangerine Peel + Dandelion [RR = 1.76, 95% CI (1.24, 2.52), *n* = 2], Acruginous Turmeric + Chinese Angelica + Astragalus + Villous Amomum [RR = 1.75, 95% CI (1.01, 3.03), *n* = 2], Liquorice + Pinellia + Tangerine Peel + Paris Polyphylla [RR = 1.74, 95% CI (1.18, 2.56), *n* = 2], and Largehead Atractylodes + Tangerine Peel + Astragalus + Yam [RR = 1.63, 95% CI (1.30, 2.05), *n* = 4]. Other sensitivity analyses were shown in Table 4.

#### 3.5.5 Tier 5: Combination of 5 herbs

The three most frequently used combination of 5 herbs were: Largehead Atractylodes + Codonopsis + Tangerine Peel + Chinese Angelica + Astragalus (n = 5), Largehead Atractylodes + Liquorice + Poria + Tangerine Peel + Astragalus (n = 5), and Largehead Atractylodes + Liquorice + Poria + Hedyotis Diffusa + Scutellaria Barbata (n = 5). The combination of 5 herbs with the two highest RR were Largehead Atractylodes + Pinellia + Acruginous Turmeric + Astragalus + White Paeony [RR = 1.67, 95% CI (1.23, 2.27), n = 3] and Largehead Atractylodes + Acruginous Turmeric + Astragalus + Wolfberry + Cuscuta [RR = 1.66, 95% CI (1.15, 2.41), n = 2]. Other sensitivity analyses were shown in Table 4.

#### 3.5.6 Tier 6: Combination of 6 herbs or above

The three most frequently used combination of 6 or more herbs were Largehead Atractylodes + Poria + Codonopsis + Tangerine Peel + Chinese Angelica + Astragalus (*n* = 4), Largehead Atractylodes + Liquorice + Codonopsis + Tangerine Peel + Chinese Angelica + Astragalus (*n* = 4), and Largehead Atractylodes + Liquorice + Poria + Codonopsis + Tangerine Peel + Astragalus (*n* = 4). Other combination of 6 or more herbs only used 2 or 3 times in the formulas. The combination of 6 or more herbs with the three highest RR were Largehead Atractylodes + Pinellia + Tangerine Peel + White Paeony + Scutellaria Barbata + Barley Sprout [RR = 2.04, 95% CI (1.37, 3.05), *n* = 2], Largehead Atractylodes + Pinellia + Tangerine Peel + Acruginous Turmeric + Astragalus + White Paeony + Scutellaria Barbata [RR = 1.86, 95% CI (1.31, 2.62), *n* = 2], and Largehead Atractylodes + Liquorice + Poria + Codonopsis + Tangerine Peel + Bupleurum [RR = 1.84, 95% CI (1.24, 2.72), *n* = 2]. Other sensitivity analyses were shown in Table 4.

### 3.6 Publication bias

**FIGURE 9:**
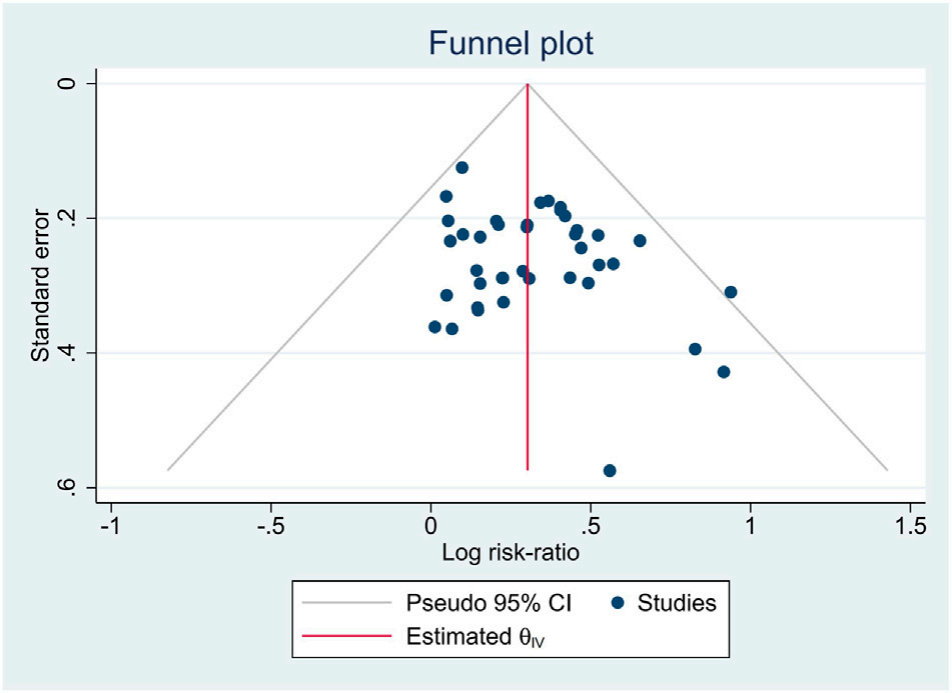
Funnel plot of publication bias.

We assessed the publication bias with a funnel plot and Egger test. The funnel plot was shown in Figure 9A *p*-value = 0.1197 in Egger test indicated that no serious publication bias was observed.

### 3.7 Quality of evidence

We assessed the quality of synthesized evidence of tumor response with GRADE approach. The results in subgroup analysis were adopted, since the subgroup of different chemotherapy regimen minimize the clinical heterogeneity. We got moderate to low quality of evidence, and the reasons to downgrade were that the majority of the evidence was from the trials with moderate methodological quality, and small sample size. The summary of findings was shown in Table 5.

**TABLE 5:** Summary of findings.

Outcome No. of participants (studies)	Relative effect (95% CI)	Anticipated absolute effects (95% CI)			Certainty
		Oxaliplatin-based chemotherapy alone (%)	CHM combined with oxaliplatin-based chemotherapy	Risk difference	
ORR (CHM + SOX vs. SOX) of participants: 1,237 (16 RCTs)	**RR 1.27** (1.15–1.40)	47.6	**60.5%** (54.8–66.7)	**12.9% more** (7.1 more to 19.1 more)	⊕⊕⊕○ Moderate^a^
ORR (CHM + FOLFOX vs. FOLFOX) No. of participants: 1,064 (15 RCTs)	**RR 1.45** (1.27–1.66)	36.2	**52.5%** (45.9–60)	**16.3% more** (9.8 more to 23.9 more)	⊕⊕⊕○ Moderate^a^
ORR (CHM + XELOX vs. XELOX) No. of participants: 772 (10 RCTs)	**RR 1.45** (1.22–1.72)	34.6	**50.1%** (42.2–59.4)	**15.5% more** (7.6 more to 24.9 more)	⊕⊕○○ Low^a^ ^,^ ^b^
DCR (CHM + SOX vs. SOX) No. of participants: 1,237 (16 RCTs)	**RR 1.10** (1.05–1.15)	78.0	**85.8%** (81.9–89.7)	**7.8% more** (3.9 more to 11.7 more)	⊕⊕⊕○ Moderate^a^
DCR (CHM + FOLFOX vs. FOLFOX) No. of participants: 1,064 (15 RCTs)	**RR 1.14** (1.08–1.21)	72.9	**83.1%** (78.8–88.2)	**10.2% more** (5.8 more to 15.3 more)	⊕⊕⊕○ Moderate^a^
ORR (CHM + XELOX vs. XELOX) No. of participants: 772 (10 RCTs)	**RR 1.12** (1.09–1.16)	69.4	**77.7%** (73.7–78.4)	**8.3% more** (6.1 more to 10.8 more)	⊕⊕⊕○ Moderate^a^

a Most information is from studies at moderate (Some concerns) risk of bias.

b Small sample size, the number of events is less than 200.

CI, confidence interval; RR, risk ratio.

Explanations.

The meaning of the bold is to highlighted the clinical value of the outcomes.

## 4 Discussion

### 4.1 Meta-analysis of trial results

Our study showed with a moderate certainty that compare with SOX, FOLFOX or XELOX regimen alone, CHM combined with SOX, FOLFOX or XELOX could significantly improve the ORR without heterogeneity, which has a practical guiding value for the clinical application of CHM synergistic chemotherapy regimen. In addition, we also found that although the relative effect of SOX regimen was lower than that of FOLFOX and XELOX, which was due to the higher anticipated absolute effects of SOX regimen alone than that of FOLFOX and XELOX, so the improvement was not particularly significant. When SOX combined with CHM, the anticipated absolute effects had increased to 60.5%, which was higher than about 50% of other regimens. The results of our study were consistent with another previously published meta-analysis of CHM combined with paclitaxel-based chemotherapy in the treatment of AGC (Li Y. et al., 2020). Compared with paclitaxel-based chemotherapy, TCM combined with paclitaxel-based chemotherapy could also significantly improve the ORR [RR = 1.39, 95% CI (1.24–1.57), I^2^ = 12%]. But the prescriptions of CHM included in the above studies are not consistent, which brings more uncertain factors to the comparison.

In reducing the incidence of AEs, CHM combined with oxaliplatin-based chemotherapy showed consistent efficacy in leukopenia, thrombocytopenia, nausea and vomiting with paclitaxel-based chemotherapy, but had more improvement advantages in anemia, gastrointestinal reactions, diarrhea, hepatorenal toxicity and peripheral neurotoxicity. It is worth noting that both paclitaxel-based and oxaliplatin-based chemotherapy are clinically useful chemotherapy regimens that easily lead to peripheral neurotoxicities (Yamashita et al., 2021). However, inconsistent clinical outcomes occurred between these regimens. CHM prescriptions in our study were effective in reducing the incidence of peripheral neurotoxicity, indicating that different herbal combinations may have different effects in reducing the incidence of AEs. Therefore, it is important to excavate the effects of herbs and their combinations which may affect the efficacy to guide clinical application. In addition, this study also found that the interventions of CHM was safe in AGC patients, and the participation of CHM did not increase the occurrence of AEs.

Furthermore, in order to explore the effect of CHM on the survival time of AGC, we reviewed the following studies and hope that will provide a useful reference, which is also the focus for future studies. A cohort study that 1:1 matched in Taiwan found that CHM could improve the OS rate of GC and reduce the risk of death by 45% (Hung et al., 2017). Afterward, using propensity score matching according to 1:3 also found that regardless of short-term or long-term application of TCM, compared with non-users, it could effectively reduce the risk of death by 41% or 59%, and pointed out that the most frequently used single herbal medicine was Astragalus (11.8%), and the most frequently used formula was Xiangsha Liujunzi Decoction (15.5%) [Codonopsis pilosula, Largehead Atractylodes, Poria, Liquorice, Muxiang, Amomum villosum] (Shih et al., 2021). Interestingly, these single herbs or formulas were generally consistent with the listed herbs in our study. In addition, a meta-analysis evaluating the efficacy of Astragalus-containing TCM combined with platinum-based chemotherapy for AGC suggested that compare with platinum-based chemotherapy alone, chemotherapy combined with Astragalus-containing TCM could improve ORR, DCR, 1 or 2-year survival rate, QoL and lower incidence of AEs (Cheng et al., 2021). These above studies strongly corroborated the credibility of the herbal compositions obtained in this study.

### 4.2 Analysis of TCM contribution

It is well-known that CHM formulas were prescriptions composed of varieties of herbs, which plays a complex and multiple effect role in clinical efficacy. Based on the characteristics of syndrome differentiation treatment, the CHM prescriptions issued by different physicians for the same disease may be different from others. Similarly, this unique treatment characteristic is significantly highlighted in this study: the specific herbs of interventions is inconsistent in clinical studies of different CHM treatments for AGC. It should be emphasized that the same herbs may be used in different studies, which provides an important basis for findings the special contribution of different herbs.

However, these herbs are not only focused on improving ORR, and perhaps some herbs pay more attention to the reduction of the occurrence of AEs to chemotherapy, or the improvement of QoL. Therefore, to eliminate the interference of other herbs, we applied sensitivity analysis to explore the effect of individual herbs on ORR in AGC one by one firstly. Then analyze the multi-herb combination containing the same herbs to further evaluate which specific herbs and their combinations are most likely to improve the ORR of chemotherapy for AGC.

Inferred to previous studies (Chen et al., 2016a; Chen et al., 2016b; Chen MH. et al., 2016), to analyze the results more cautiously, our selected herbs and compositions must have more significant differences, no heterogeneity, and equal or higher RRs within each level. Based on the above criteria, from the results of multiple sensitivity analyses, we identified seven herbs as potential effect of synergistic action with chemotherapy for AGC: Astragalus (*n* = 13), Liquorice (*n* = 23), Poria (*n* = 21), Largehead Atractylodes (*n* = 26), Chinese Angelica (*n* = 14), Codonopsis (*n* = 18), and Tangerine Peel (*n* = 17). Among them, Dandelion and Paris Polyphylla possessed the highest effect estimate (RR = 1.47) in individual herb analysis. In addition, this study also analyzed the combination of herbs. It is reported that the composition of Codonopsis and Liquorice, and the composition of Codonopsis, Liquorice, Largehead Atractylodes and Poria both significantly improved the ORR of TCM combined with paclitaxel-based chemotherapy, which also provided supporting evidence for our study (Li Y. et al., 2020).

Surprisingly, some relatively high-frequency herbs such as Pinellia (*n* = 20), Acruginous Turmeric (*n* = 15), or some herbs with relatively high RR such as Dandelion [1.47 (1.17, 1.83)], Paris Polyphylla [1.47 (1.07, 2.02)], and Red Paeony [1.46 (1.13, 1.88)] in the individual herb analysis were not included in the final combination. We found that compared to the ORR under multi-herb combinations, the above potential herbs may not show a consistent effect with other herbs under multi-herb combinations. Nevertheless, these herbs also have the potential to contribute to ORR, and we found that these individual herbs with relatively high RR were mostly combined with the above 7 herbs in multi-herb combinations showing higher ORR value. Therefore, these herbs can also be considered for subsequent development.

In addition, we also excluded some highly heterogeneous herbs, such as Panax notoginseng (I^2^ = 73.56%), although commonly used in AGC, which seems to significantly reduce ORR [1.71, (0.84, 3.48)], but only two studies were evaluated. Notably, no negative RRs emerged for any of the herbs or combinations, suggesting that these herbs did not impair the effects of chemotherapy.

### 4.3 Strength and limitations

To our knowledge, this is the first meta-analysis evaluating the efficacy and safety of CHM combined with oxaliplatin-based chemotherapy in AGC. Our study suggests that the synergistic treatment of CHM may be more TRR-improving on FOLFOX and XELOX regimens, and has high safety for clinical application. In addition, this study complied strict inclusion and exclusion criteria to exclude studies without a clear randomization method, which reduces the RoB in studies and improves the reliability of results. Therefore, quality of only one outcome was classified as low, and other qualities of evidence were moderate. There was also no serious publication bias.

At the same time, our study also has several limitations. Firstly, lacks multi-center, large-sample RCTs and some of the original study sample sizes are small, which causes bias in the results, which requires the publication of more high-quality clinical studies. Secondly, the included studies had an insufficient assessment of long-term survival indicators (OS, PFS), and our study showed that CHM could improve TRR in AGC, but whether it could translate into a benefit in long-term outcomes, also requires reassessment. In addition, CHMs are a part of TCM preparations, including Chinese patent medicines and TCM injections, and these preparation types should also be widely evaluated. Even if there have been studies on the anti-tumor efficacy of TCM preparations such as cinobufotalin (Sun et al., 2019) and brucea javanica oil injection (Wang et al., 2021) for AGC. A more comprehensive evaluation of CHM is needed to obtain more real, effective and safe evidence.

In addition, an obvious innovation point lies in exploring special herbs that have a synergistic action with chemotherapy on the ORR for AGC. The advantage of this methodology is based on the contribution of each herb to the contribution of ORR and is not simply based on the total frequency of herb emergence. A sensitivity analysis was chosen to identify the contribution of each herb in the study to ORR without missing it at a lower frequency. In summary, one cannot simply choose according to the overall frequency or the order of effect sizes of herbs in the dataset, but rather needs to consider the overall effect sizes at multiple levels simultaneously. For the selection process of final results, these herbs do not show improvement in ORR in only one clinical intervention study. In contrast, these herbs have shown consistent or higher effects in multiple studies and multiple combinations. Another limitation is that all possible combinations cannot be evaluated, and as the number of multi-herb combinations increases, the number of combinations with a significant difference and no heterogeneity is smaller, the number of corresponding clinical studies and their sample size is also reduced, and the interpretation of the results should be more cautious. And there is no clear clinical trial to evaluate the clinical efficacy of this formula, and the corresponding RCT should be carried out for evaluation in the future.

### 4.4 Review of anti-gastric cancer mechanisms of seven selected herbs

Similarly, we cannot clarify that the listed herbs are all for improving the efficacy of chemotherapy, however, seven herbs have been used for AGC treatment in clinical in China (Cao et al., 2009). Among them, four herbs constitute a typical formula in China, Sijunzi decoction, which has been shown to reduce the nuclear accumulation and DNA-binding activity of β-catenin, thereby repressing cell growth and inducing apoptosis in human GC MKN74 and MKN45 cells (Li et al., 2021). Network pharmacology and experimental validation suggested that Sijunzi decoction could inhibit tumor proliferation and angiogenesis by down-regulating the expression of VEGFA, iNOS, COX-2, and Bax/Bcl2 proteins in NCG-bearing mice with human gastric adenocarcinoma cell NUGC-4, regulate the PI3K/AKT pathway, and induce apoptosis (Ding et al., 2022). To further explore how seven herbs improve the short-term efficacy of platinum-based chemotherapy for AGC, we will review each herb to assess the mechanistic evidence of the specific anti-tumor activity. We found that the number of published mechanistic studies of these herbs was uneven, with Astragalus and Liquorice being more intensively studied.

#### 4.4.1 Astragalus

Previous study has shown that Astragalus dose-dependently stimulates dendritic cells (DCs) to express Toll-like receptor 4 (TLR4), thereby enhancing cellular immune function, and inhibiting IκB-α protein expression and regulating NF-kB signaling pathways (Tian et al., 2014). Secondly, after co-culture with MKN45 *in vitro*, MTT showed that Astragalus could reduce cell viability and induce apoptosis, and significantly reduce the number of cells. *In vitro* experiments also confirmed that Astragalus could significantly inhibit tumor diameter and weight, with a tumor inhibition rate of 57.1%. Another study also found that the aqueous extract of Astragalus could mediate antigen-presenting cells (APCs) to stimulate CXCR5 + Tfh-like cells to highly express IL-21, enhance humoral immunity and regulate CD8 + T cell activity (Dong et al., 2021).

In addition, the pharmacologically active components of Astragalus include polysaccharides, saponins and flavonoids, of which Astragalus polysaccharide (APS) is the most widely studied (Ma et al., 2002). In one study evaluating the anti-tumor activity of APS combined with adriamycin in human GC cell SGC-7901 and SGC-7901/ADR, the results showed that APS reduced cell viability and enhanced the rate of apoptosis in a time-dosage dependent manner, up-regulated p-AMPK and caspase-3 expression levels, induced apoptosis through the AMPK pathway, and improved the sensitivity of GC cells to adriamycin, suggesting that APS can be used as a chemosensitizer (Song et al., 2020). This paper speculated that the effect may be related to the decreased expression of MDR1, and the overexpression of tumor suppressor genes such as SEMA3F, P21^WAF1/CIP1^ and FBXW7. Similarly, APS can also inhibit the expression of phosphorylated AKT (p-AKT) and MMP-9, and then inhibit the proliferation, migration and invasion of GC AGS cells through the AKT pathway, and also induce autophagy (Wu et al., 2018). In addition, APS also has a variety of immunoregulatory activities, such as regulating B lymphocytes and cytokines, especially inducing the activation and differentiation of splenic DCs, followed by the completion of Th2 to Th1 transition and enhancing the immune function of T lymphocytes (Liu et al., 2011).

#### 4.4.2 Liquorice

A large number of bioactive components can be isolated from Liquorice, including triterpenoid saponins, flavonoids, isoflavones and chalcone (Asl et al., 2008). Glycyrrhizic acid (GA) is usually considered to be the main active component. The study has shown that GA can induce G1/S phase arrest of cell cycle and apoptosis by down-regulating the expression of cyclin D1, D2, D3, E1, E2, Bcl-2, survivin and p65, up-regulating the protein levels of Bax, PARP and pro-caspase-3, -8, -9. The proliferation of human GC cells (MGC-803, BGC-823, SGC-7901) has a time- and dose-dependent inhibitory effect by down-regulating PI3K/AKT signaling pathway (Wang et al., 2020).

Isoliquiritigenin (ISL) is a flavonoid extracted from Liquorice. Studies have shown that ISL can down-regulate Bcl-2 protein and p62, up-regulate Bax protein, caspase-3, Beclin 1 and LC3II/LC3I ratio, mediate apoptosis and autophagy of human GC MKN28 cells by inhibiting PI3K/AKT/mTOR signaling pathway, thereby inhibiting cell proliferation, and reducing invasion and migration (Zhang et al., 2018). ISL can also induce apoptosis in human GC MGC-803 cells by increasing free calcium concentration and decreasing mitochondrial transmembrane potential (Ma et al., 2001). However, licochalcone A (LCA), as a cytotoxic flavonoid, alone or in combination with 5-FU, increased the expression levels of Bax, PARP, tumor proteins 21, 27, 53 and caspase 3, and decreased the expression levels of Bcl-2, cyclin A, cyclin B and MDM2, which in turn blocked G 2/M cell cycle progression and induced apoptosis, inhibiting SGC7901 and MKN-45 cell proliferation in a time-dependent manner (Xiao et al., 2011; Lin et al., 2017). In addition, LCA can also increase the level of reactive oxygen species (ROS) and induce oxidative stress and apoptosis of BGC-823 cells through PI3K/AKT/mTOR and MAPK signaling cascades (Hao et al., 2015).

In addition, glycyrrhetinic acid (GA) can inhibit the viability of GC cells in a dose- and time-dependent manner, but its toxic and side effects limit its wide application, thus making many beneficial explorations on related derivatives (Xu et al., 2017). For example, 11-deoxy glycyrrhetinic acid can effectively inhibit GC formation by up-regulating p21 and down-regulating cdc2 and cyclin B1, mediating BID translocation from the nucleus to mitochondria, thereby inducing apoptosis and G2 phase arrest in GC cell (Lin et al., 2014). For another example, 18β-glycyrrhetinic acid (18β-GA) can inhibit MMP-2 and MMP-9 activities in a dose-dependent manner, up-regulate E-cadherin expression and down-regulate vimentin expression, and reduce the metastatic potential of human GC cell line SGC-7901 cells through ROS/PKC-α/ERK signaling pathway and inhibition of EMT (Cai et al., 2018). In addition, new isoliquiritigenin (ISL) analogues (Huang et al., 2020), licochalcone A derivatives (LCA) (Shibata, 1994) can also provide more options for anti-GC drug research and development.

#### 4.4.3 Poria

*In vitro* studies confirmed that Poria combined with oxaliplatin significantly decreased Snail, Twist, vimentin, and N-cadherin mRNA and protein expression, significantly increased E-cadherin mRNA and protein expression, inhibited the EMT process, and decreased the invasion and migration of SGC7901 (Wang N. et al., 2018). This study also found that Poria could reduce the tumor volume, and improve the morphological parameters of GC cells. In addition, both dehydroebriconic acid and dehydrotrametenonic acid found from Poria sclerotia inhibited the growth of GC cells by arresting the G1 phase of the cell cycle, with LD (50) values of 63.6 and 38.4 microM, respectively (Mizushina et al., 2004).

#### 4.4.4 Largehead Atractylodes

Atractylenolide (AT) is the main anticancer active ingredient of Largehead Atractylodes. Atractylenolide I (AT-I) has been found to inactivate the Notch signaling pathway by down-regulating the protein and mRNA expression of Notch1, Jagged1, Hes1, Hey1 and CD44, inhibiting the sphere formation ability and cell viability of HGC-27, MGC-803 and MKN-45, thereby inhibiting the self-renewal ability and cell proliferation of GC stem-like cells (GCSC) and inducing apoptosis (Ma et al., 2014). A randomized controlled trial also verified the therapeutic effect of AT-1 on GC cachexia (Liu et al., 2008). Similarly, for Atractylenolide II (AT-II), Bax expression could also be up-regulated, and B-cell lymphoma 2 (Bcl-2), phosphorylated protein kinase B (p-Akt), and phosphorylated ERK (p-ERK) expression could be down-regulated, which induced apoptosis and inhibited proliferation and migration of HGC-27 and AGS in a concentration- and time-dependent manner by inactivating Ras/ERK and PI3K/Akt signaling pathways (Tian et al., 2017). AT-1 and AT-2 also inhibit Akt/IκBα/NF-κB signaling pathway to play a role, thereby inhibiting gastritis to GC transformation (Amin et al., 2022). In addition, the aqueous extract of Largehead Atractylodes could inhibit the proliferation of BGC-823 and SGC-7901, decrease the mitochondrial transmembrane potential, and induce apoptosis and cell cycle arrest in a dose- and time-dependent manner (Zhao et al., 2014).

#### 4.4.5 Chinese Angelica

Decursin is one of the active components of Chinese Angelica, which has been confirmed to down-regulate CXC chemokine receptor 7 (CXCR7) and Bcl-2 expression in a dose-dependent manner, mediate STAT3/c-Myc signaling pathway, induce apoptosis, and inhibit the proliferation, migration, and invasion of SNU484 and SNU216 (Kim et al., 2019). Another study by the same team also confirmed that Decursin could reduce cell viability, inhibited cell growth and induce G0/G1 arrest *in vitro* in a dose- and time-dependent manner (Kim et al., 2021). And by promoting the accumulation of LC3 and SQSTM1, inhibiting CTSC and E2F3 expression, and reducing the activity of lysosomal protein cathepsin C (CTSC), thereby inducing autophagic flux disorders. While *in vivo* studies, Decursin decreased the growth of tumor spheroids and patient-derived gastric organoids, and regulated the expression of CTSC and autophagy-related proteins, which in turn validated the *in vivo* experimental results.

In addition, a clinical data from Taiwan verified that Chinese Angelica could prolong the survival rate of GC patients [adjusted hazard ratio (HR) 0.72 [95% CI, 0.57–0.92] (*p* = 0.009)], and experimental studies also found that N-butylidenephthalide (BP), the active component of Chinese Angelica, could induce increased REDD1 expression, inhibit mTOR signaling, activate apoptosis through the mitochondrial pathway, and inhibit the proliferation and EMT of AGS, NCI-N87, and TSGH-9201 cells (Liao et al., 2018).

#### 4.4.6 Codonopsis

Lobetyolin (LBT) and Lobetyol are the essential components of Codonopsis. The anti-GC activity of LBT mainly depends on the regulation of glutamine metabolism, the decrease of mRNA and protein expression of amino acid transporter alanine-serine-cysteine transporter 2 (ASCT2), the induction of apoptosis and the inhibition of GC cell proliferation (Bailly, 2021). Lobetyol induced apoptosis and G1/S phase cell cycle arrest in MKN45 cells by mediating MAPK signaling pathway in a time- and dose-dependent manner (Shen et al., 2016). Both can be further considered as potential anti-GC active ingredients. In addition, the Chinese medicine formula “Weikang Keli”, containing Codonopsis and Largehead Atractylodes, can induce autophagic cell death in SGC-7901 cels. *In vitro* experiments showed that compared with the positive control of 5-FU, “Weikang Keli” aqueous extract could reduce the tumor volume in the GC model. Although the difference was not statistically significant, the motility, response sensitivity and food intake were increased (Huo et al., 2013).

#### 4.4.7 Tangerine Peel

Nobiletin (Nob), a poly methoxy flavonoid extracted from Tangerine Peel, has been shown to inhibit MMP-9 activity in a concentration-dependent manner and to significantly reduce the total weight and number of disseminated nodules in a SCID mouse model (0.07g vs. 0.78g, *p* = 0.0059; 7.5 vs. 69.3/body, *p* = 0.0001), thereby inhibiting the proliferation of TMK-1 cell with peritoneal disseminated nodule formation, which is beneficial in preventing GC metastasis (Minagawa et al., 2001). In addition, it could also up-regulate E-cadherin protein expression and down-regulate vimentin, fibronectin, MMP-9 protein and p-STAT3 expression in SGC-7901 cell, by inhibiting the STAT3 pathway, followed by inhibiting EMT (Yang et al., 2020).

In addition, Nob-induced apoptosis is mediated by activating ER stress, decreasing phosphorylated Akt and mTOR, and mediating protective autophagy, thereby inhibiting GC proliferation in SNU-16 cells (Moon et al., 2016). And it increased Bax/Bcl-2 ratio, caspase-3/9 proteolytic activation with degradation of poly (ADP-ribose) polymerase (PARP) protein, induced apoptosis and had a synergistic anticancer effect with 5-fluorouracil in p53-mutant SNU-16 cell (Moon et al., 2013).

## 5 Conclusion

Overall, CHM has a positive effect on improving oxaliplatin-based chemotherapy for AGC, which can improve short-term efficacy and reduce the incidence of AEs. In the sensitivity analysis of herbal medicines, it was found that herbal medicines such as Astragalus, Liquorice, Poria, Largehead Atractylodes, Chinese Angelica, Codonopsis, and Tangerine Peel had more advantages in increasing ORR. Previous mechanistic studies also confirmed their anti-GC activity, and the results of our study will provide beneficial evidence support for the combination therapy of CHM. Considering factors such as low quality of literature and insufficient sample size for clinical trials corresponding to shortlisted herbal medicines, quality of evidence was not high, definite conclusions cannot be drawn. More rigorously designed, large-sample, multi-center RCTs of herbal synergistic chemotherapy are needed in the future to better validate the action characteristics of CHM and its potential herbal medicines.
