# Supplementary material for: Chinese herbal medicine combined with oxaliplatin-based chemotherapy for advanced gastric cancer: A systematic review and meta-analysis of contributions of specific medicinal materials to tumor response
Source: Front Pharmacol. 2022 Aug 25;13:977708. doi: 10.3389/fphar.2022.977708 (PMC9453215; doi:10.3389/fphar.2022.977708)
Supplement: Supplementary file 1 [file DataSheet1.DOCX]

Supplementary File 1. PRISMA-2009 Checklist

| **Section/topic** | **#** | **Checklist item** | **Reported on page #** |
| --- | --- | --- | --- |
| **TITLE** | | |  |
| Title | 1 | Identify the report as a systematic review, meta-analysis, or both. | Page 1 |
| **ABSTRACT** | | |  |
| Structured summary | 2 | Provide a structured summary including, as applicable: background; objectives; data sources; study eligibility criteria, participants, and interventions; study appraisal and synthesis methods; results; limitations; conclusions and implications of key findings; systematic review registration number. | Page 2-3 |
| **INTRODUCTION** | | |  |
| Rationale | 3 | Describe the rationale for the review in the context of what is already known. | Page 4-7 |
| Objectives | 4 | Provide an explicit statement of questions being addressed with reference to participants, interventions, comparisons, outcomes, and study design (PICOS). | Page 7 |
| **METHODS** | | |  |
| Protocol and registration | 5 | Indicate if a review protocol exists, if and where it can be accessed (e.g., Web address), and, if available, provide registration information including registration number. | Page 8 |
| Eligibility criteria | 6 | Specify study characteristics (e.g., PICOS, length of follow-up) and report characteristics (e.g., years considered, language, publication status) used as criteria for eligibility, giving rationale. | Page 8-9 |
| Information sources | 7 | Describe all information sources (e.g., databases with dates of coverage, contact with study authors to identify additional studies) in the search and date last searched. | Page 9 |
| Search | 8 | Present full electronic search strategy for at least one database, including any limits used, such that it could be repeated. | Supplementary File 2 |
| Study selection | 9 | State the process for selecting studies (i.e., screening, eligibility, included in systematic review, and, if applicable, included in the meta-analysis). | Page 9 |
| Data collection process | 10 | Describe method of data extraction from reports (e.g., piloted forms, independently, in duplicate) and any processes for obtaining and confirming data from investigators. | Page 9-10 |
| Data items | 11 | List and define all variables for which data were sought (e.g., PICOS, funding sources) and any assumptions and simplifications made. | Page 9-10 |
| Risk of bias in individual studies | 12 | Describe methods used for assessing risk of bias of individual studies (including specification of whether this was done at the study or outcome level), and how this information is to be used in any data synthesis. | Page 10 |
| Summary measures | 13 | State the principal summary measures (e.g., risk ratio, difference in means). | Page 10-11 |
| Synthesis of results | 14 | Describe the methods of handling data and combining results of studies, if done, including measures of consistency (e.g., I^2^) for each meta-analysis. | Page 10 |
| Risk of bias across studies | 15 | Specify any assessment of risk of bias that may affect the cumulative evidence (e.g., publication bias, selective reporting within studies). | Page 10 |
| Additional analyses | 16 | Describe methods of additional analyses (e.g., sensitivity or subgroup analyses, meta-regression), if done, indicating which were pre-specified. | Page 10-11 |
| **RESULTS** | | |  |
| Study selection | 17 | Give numbers of studies screened, assessed for eligibility, and included in the review, with reasons for exclusions at each stage, ideally with a flow diagram. | Page 11-12 Figure 1 |
| Study characteristics | 18 | For each study, present characteristics for which data were extracted (e.g., study size, PICOS, follow-up period) and provide the citations. | Page 12-13  Table 1-2 |
| Risk of bias within studies | 19 | Present data on risk of bias of each study and, if available, any outcome level assessment (see item 12). | Page 13-14  Figure 2 |
| Results of individual studies | 20 | For all outcomes considered (benefits or harms), present, for each study: (a) simple summary data for each intervention group (b) effect estimates and confidence intervals, ideally with a forest plot. | Page 14-21  Figure 3-8 |
| Synthesis of results | 21 | Present results of each meta-analysis done, including confidence intervals and measures of consistency. | Page 14-21  Figure 3-8 |
| Risk of bias across studies | 22 | Present results of any assessment of risk of bias across studies (see Item 15). | Page 25  Figure 9 |
| Additional analysis | 23 | Give results of additional analyses, if done (e.g., sensitivity or subgroup analyses, meta-regression [see Item 16]). | Page 21-26 |
| **DISCUSSION** | | |  |
| Summary of evidence | 24 | Summarize the main findings including the strength of evidence for each main outcome; consider their relevance to key groups (e.g., healthcare providers, users, and policy makers). | Page 26-29 |
| Limitations | 25 | Discuss limitations at study and outcome level (e.g., risk of bias), and at review-level (e.g., incomplete retrieval of identified research, reporting bias). | Page 30-31 |
| Conclusions | 26 | Provide a general interpretation of the results in the context of other evidence, and implications for future research. | Page 38 |
| **FUNDING** | | |  |
| Funding | 27 | Describe sources of funding for the systematic review and other support (e.g., supply of data); role of funders for the systematic review. | Page 39 |

Supplementary File 2. Search strategy used in PubMed

#1 Stomach Neoplasms[MeSH Terms]

#2 (((((((((((((((((Neoplasm, Stomach[Title/Abstract]) OR (Stomach Neoplasm[Title/Abstract])) OR (Neoplasms, Stomach[Title/Abstract])) OR (Gastric Neoplasms[Title/Abstract])) OR (Gastric Neoplasm[Title/Abstract])) OR (Neoplasm, Gastric[Title/Abstract])) OR (Neoplasms, Gastric[Title/Abstract])) OR (Cancer of Stomach[Title/Abstract])) OR (Stomach Cancers[Title/Abstract])) OR (Gastric Cancer[Title/Abstract])) OR (Cancer, Gastric[Title/Abstract])) OR (Cancers, Gastric[Title/Abstract])) OR (Gastric Cancers[Title/Abstract])) OR (Gastric Carcinoma[Title/Abstract])) OR (Stomach Cancer[Title/Abstract])) OR (Cancer, Stomach[Title/Abstract])) OR (Cancers, Stomach[Title/Abstract])) OR (Cancer of the Stomach[Title/Abstract])

#3 #1 OR #2

#4 (((((((Advanced[Title/Abstract]) OR (IV stage[Title/Abstract])) OR (Ⅳ Stage[Title/Abstract])) OR (Metastatic[Title/Abstract])) OR (Metastasis[Title/Abstract])) OR (Metastases[Title/Abstract])) OR (Stage IV[Title/Abstract])) OR (Stage Ⅳ[Title/Abstract])

#5 ((Herbal Medicine[MeSH Terms]) OR (Medicine, Chinese Traditional[MeSH Terms])) OR (Drugs, Chinese Herbal[MeSH Terms])

#6 (((((((((((((Herbal Medicine[Title/Abstract]) OR (Medicine, Herbal[Title/Abstract])) OR (Medicine, Chinese Traditional[Title/Abstract])) OR (Traditional Chinese Medicine[Title/Abstract])) OR (Traditional Medicine, Chinese[Title/Abstract])) OR (Chinese Traditional Medicine[Title/Abstract])) OR (Chinese Medicine, Traditional[Title/Abstract])) OR (Drugs, Chinese Herbal[Title/Abstract])) OR (Chinese Drugs, Plant[Title/Abstract])) OR (Chinese Herbal Drugs[Title/Abstract])) OR (Herbal Drugs, Chinese[Title/Abstract])) OR (Plant Extracts, Chinese[Title/Abstract])) OR (Chinese Plant Extracts[Title/Abstract])) OR (Extracts, Chinese Plant[Title/Abstract])

#7 #5 or #6

#8 (Antineoplastic Combined Chemotherapy Protocols[MeSH Terms]) OR (Oxaliplatin[MeSH Terms])

#9 (((((((((((((((((((((((((Antineoplastic Combined Chemotherapy[Title/Abstract]) OR (Folfox protocol[Title/Abstract])) OR (FOLFOX4 protocol[Title/Abstract])) OR (FOLFOX-6 protocol[Title/Abstract])) OR (FOLFOX-7 protocol[Title/Abstract])) OR (Folfox regimen[Title/Abstract])) OR (FOLFOX-4 protocol[Title/Abstract])) OR (FOLFOX4[Title/Abstract])) OR (FOLFOX-4[Title/Abstract])) OR (FOLFOX-6[Title/Abstract])) OR (FOLFOX6[Title/Abstract])) OR (FOLFOX-7[Title/Abstract])) OR (FOLFOX7[Title/Abstract])) OR (XELOX[Title/Abstract])) OR (CAPEOX[Title/Abstract])) OR (SOX[Title/Abstract])) OR (Oxaliplatin[Title/Abstract])) OR (L-OHP Cpd[Title/Abstract])) OR (Oxaliplatine[Title/Abstract])) OR (Eloxatine[Title/Abstract])) OR (Eloxatin[Title/Abstract])) OR (ACT 078[Title/Abstract])) OR (ACT-078[Title/Abstract])) OR (ACT078[Title/Abstract])) OR (Oxaliplatin based chemotherapy[Title/Abstract])) OR (Oxaliplatin based chemotherapies[Title/Abstract])

#10 #8 OR #9

#11 ((randomized controlled trial[pt] OR controlled clinical trial[pt] OR randomized[tiab] OR placebo[tiab] OR clinical trials as topic[mesh:noexp] OR randomly[tiab] OR trial[ti] NOT (animals[mh] NOT humans [mh])))

#12 #3 AND #4 AND #7 AND #10 AND #11

Supplementary File 3. Summary of all of the included trails

| Study | Formulation | Source | Species, concentration | Quality control reported? (Y/N) | Chemical analysis reported?  (Y/N) |
| --- | --- | --- | --- | --- | --- |
| Bao PQ (2020 | Yiqi Huoxue Formula | - | ·Dried roots of *Curcuma phaeocaulis* Val., 10g  ·Dried roots of *Angelica sinensis* (Oliv.) Diels, 10g  ·Fruits of *Citrus medica* L. var. sarco- dactylis Swingle, 10g  ·Fruits of *Amomum villosum* Lour., 10g  ·Dried roots and rhizomes of *Panax noto·Dried roots and rhizomes of Panax ginseng C. A. Mey.* (Burk.) F. H. Chen, 10g  ·Dried roots of *Codonopsis pilosula* (Franch.) Nannf., 20g  ·Dried roots of *Astragalus mongholicus* Bunge., 30g | N | N |
| Cai JY 2019 | Jianpi Yiqi Formula | - | ·Dried roots of *Codonopsis pilosula* (Franch.) Nannf.,15g  ·Dried sclerotia of *Poria cocos* (Schw.) Wolf, 12g  ·Dried rhizomes of *Atractylodes macrocephala* Koidz., 15g  ·Leaf of *Perilla frutescens* (L.) Britt., 10g  ·Seed of *Coix lacryma-jobi* L., 15g  ·Dried rhizomes of *Pinellia ternata* (Thunb.) Makino., 10g  ·Dried flowers of *Inula japonica* Thunb., 15g  ·Ruddle, 30g  ·Fruits of *Amomum villosum* Lour., 6g  ·Dried roots and rhizomes of *Glycyrrhiza uralensis* Fisch., 6g | N | N |
| Chen K 2007 | TCM syndrome differentiation Formula 1;  TCM syndrome differentiation Formula 2;  TCM syndrome differentiation Formula 2 | - | TCM syndrome differentiation Formula 1:  ·Dried herbs of *Pogostemon cablin* (Blanco) Benth., 10g  ·Dried stems of *Perilla frutescens* (L.) Britt., 10g  ·Dried rhizomes of *Pinellia ternata* (Thunb.) Makino., 10g  ·Dried peels of *Citrus reticulata* Blanco, 10g  ·Dried roots of *Aucklandia lappa* Decne., 10g  ·Dried sclerotia of *Poria cocos* (Schw.) Wolf, 20g  ·Dried fruits of *Citrus aurantium* L., 10g  ·Membrane of Chickens Gizzard, 10g  ·Dried roots and rhizomes of *Glycyrrhiza uralensis* Fisch., 6g；  TCM syndrome differentiation Formula 2:  ·Dried roots of *Astragalus mongholicus* Bunge., 30g  ·Dried roots of *Pseudostellaria heterophylla* (Miq.) Pax, 15g  ·Dried roots of *Codonopsis pilosula* (Franch.) Nannf., 15g  ·Dried sclerotia of P*oria cocos* (Schw.) Wolf, 30g  ·Dried rhizomes of *Atractylodes macrocephala* Koidz., 15g  ·Seed of *Coix lacryma-jobi* L., 30g  ·Dried peels of *Citrus reticulata* Blanco, 10g  ·Membrane of Chickens Gizzard, 10g  ·Dried rhizomes of *Polygonatum kingianum* Coll.et Hemsl., 10g  ·Dried fruits of *Lycium barbarum* L., 15g  ·Dried seeds of *Cuscuta chinensis* Lam., 15g  ·Dried fruits of *Ligustrum lucidum* Ait., 15g  ·Dried fruits of *Psoralea corylifolia* L., 10g  ·Dried herbs of Scutellaria barbata D.Don, 15g  ·Dried herbs of *Hedyotis diffusa* Willd., 15g  ·Dried roots of *·*Dried rhizomes of *Paris polyphylla* Smith var.yunnanensis(Franch.)Hand.-Mazz. Smith var.yunnanensis (Franch.) Hand.-Mazz., 15g  ·Dried roots of *Sophora flavescens* Ait., 30g  ·Dried roots and stems of *Cudrania tricuspidata* (Carr.) Bur. ex Lavallee, 30g  ·Dried rhizomes of *Ligusticum chuanxiong* Hort., 15g  ·Dried fruits of *Akebia quinata* (Thunb.) Decne., 30g  ·Dried herbs of *Salvia chinensis* Benth., 30g；  TCM syndrome differentiation Formula 3:  ·Dried roots of *Astragalus mongholicus* Bunge., 30g  ·Dried roots of *Pseudostellaria heterophylla* (Miq.) Pax, 15g  ·Dried roots of *Codonopsis pilosula* (Franch.) Nannf., 15g  ·Dried sclerotia of *Poria cocos* (Schw.) Wolf, 20g  ·Dried sclerotia of *Polyporus umbellatus* (Pers.) Fries, 20g  ·Dried rhizomes of *Atractylodes macrocephala* Koidz., 15g  ·Seed of *Coix lacryma-jobi* L., 30g  ·Membrane of Chickens Gizzard, 10g  ·Dried fruits of *Lycium barbarum* L., 15g  ·Dried seeds of *Cuscuta chinensis* Lam., 10g  ·Dried fruits of *Ligustrum lucidum* Ait., 15g  ·Dried fruits of *Cornus officinalis* Sieb. et Zucc., 15g  ·Dried body of *Hericium erinaceus* (Bull. ex Fr.) Pers., 30g  ·Dried herbs of ·Dried roots of *Scutellaria baicalensis Georgi barbata* D.Don, 30g  ·Dried herbs of *Hedyotis diffusa* Willd., 30g  ·Dried roots of ·Dried rhizomes of *Paris polyphylla Smith var*.yunnanensis(Franch.)Hand.-Mazz. Smith var.yunnanensis (Franch.) Hand.-Mazz., 30g  ·Dried rhizomes of ·Dried rhizomes of *Smilax china* L. china L., 30g  ·Dried roots of *Ranunculus ternatus* Thunb., 30g  ·Dried herbs of *Salvia chinensis* Benth., 30g  ·Dried fruits of *Akebia quinata* (Thunb.) Decne., 30g  ·Dried roots and stems of *Cudrania tricuspidata* (Carr.) Bur. ex Lavallee, 30g  ·Dried rhizomes of *Dioscorea bulbifera* L., 10g  ·Shell of *Ostrea gigas* Thunberg, 30g  ·Dried body of *Sargassum pallidum* (Turn.) C.Ag., 30g | N | N |
| Chi HC 2010 | Yiqi Huoxue Formula | - | ·Dried roots of *Astragalus mongholicus* Bunge., 15g  ·Dried roots of *Pseudostellaria heterophylla* (Miq.) Pax, 30g  ·Dried stems of *Spatholobus suberectus* Dunn, 30g  ·Dried rhizomes of *Atractylodes macrocephala* Koidz., 10g  ·Dried sclerotia of *Poria cocos* (Schw.) Wolf, 10g  ·Dried fruits of *Lycium barbarum* L., 15g  ·Dried fruits of *Ligustrum lucidum* Ait., 15g  ·Dried seeds of *Cuscuta chinensis* Lam., 15g  ·Dried roots of *Paeonia lactiflora* Pall., 10g  ·Seed of *Coix lacryma-jobi* L., 30g  ·Dried roots of *Actinidia chinensis Planch*. var. hispida C.F.Liang.15g | N | N |
| Chu RG 2017 | Zhangshi Yiwei Decoction | Prepared by Affiliated Hospital of Jiangxi University of Chinese Medicine | ·Dried rhizomes of *Atractylodes macrocephala* Koidz., 10g  ·Dried sclerotia of *Poria cocos* (Schw.) Wolf, 15g  ·Dried roots of *Codonopsis pilosula* (Franch.) Nannf.,20g  ·Dried rhizomes of *Coptis chinensis* Franch., 6g  ·Dried peels of *Citrus reticulata* Blanco, 10g  ·Dried bulbus of *Fritillaria thunbergii* Miq., 10g  ·Dried roots of *Aucklandia lappa* Decne., 6g  ·Dried rhizomes of *Pinellia ternata* (Thunb.) Makino., 10g  ·Fruits of *Amomum villosum* Lour., 6g  ·Dried herbs of *Taraxacum mongolicum* Hand.-Mazz., 15g  ·Dried barks of *Albizia julibrissin* Durazz., 15g  ·Dried rhizomes of *Bletilla striata* (Thunb.) Reichb.f., 20g  ·Dried roots and rhizomes of *Glycyrrhiza uralensis* Fisch., 6g | N | N |
| Feng TM 2021 | Xuezheng Decoction | - | ·Dried rhizomes of *Curcuma phaeocaulis* Val., 15g  ·Dried herbs of *Hedyotis diffusa* Willd., 15g  ·Dried rhizomes of *Smilax china* L., 15g  ·Dried roots of *Angelica sinensis* (Oliv.) Diels, 10g  ·Dried roots of *Paeonia lactiflora* Pall., 10g  ·Dried rhizomes of *Sparganium stoloniferum* Buch.-Ham., 10g  ·Dried twigs of *Cinnamomum cassia* Presl, 5g  ·Resin of *Boswellia carterii* Birdw., 5g  ·Resin of *Commiphora myrrha* Engl., 5g | N | N |
| Feng YL 2017 | Gancao Xiexin Decoction | Prepared by Hospital of Chengdu University of Traditional Chinese Medicine | ·Dried roots and rhizomes of *Glycyrrhiza uralensis* Fisch., 15g  ·Dried roots of *Scutellaria baicalensis* Georgi, 15g  ·Dried rhizomes of *Coptis chinensis* Franch., 10g  ·Dried rhizomes of *Pinellia ternata* (Thunb.) Makino., 15g  ·Dried rhizomes of *Zingiber officinale* Rosc.,10g  ·Dried roots and rhizomes of *Panax ginseng* C. A. Mey., 30g  ·Dried fruits of *Ziziphus jujuba* Mill.,15g | N | N |
| Gong M 2020 | Wenyang Sanjie Decoction | - | ·Dried roots of *Astragalus mongholicus* Bunge., 30g  ·Dried rhizomes of *Dioscorea opposita* Thunb., 30g  ·Dried roots and rhizomes of *Clematis chinensis* Osbeck, 30g  ·Seed of *Coix lacryma-jobi* L., 30g  ·Dried herbs of *Scutellaria barbata* D.Don, 30g  ·Dried roots of *Paeonia lactiflora* Pall., 30g  ·Ruddle, 30g  ·Dried rhizomes of *Atractylodes macrocephala* Koidz., 20g  ·Dried fruits of *Psoralea corylifolia* L., 20g  ·Dried seeds of *Cuscuta chinensis* Lam., 20g  ·Dried sprouts of *Hordeum vulgare* L. , 20g  ·Dried herbs of *Taraxacum mongolicum* Hand.-Mazz., 20g  ·Dried fruits of *Lycium barbarum* L., 20g  ·Dried rhizomes of *Curcuma phaeocaulis* Val., 15g  ·Membrane of *Chickens Gizzard*, 15g  ·Dried flowers of *Inula japonica* Thunb., 15g  ·Dried rhizomes of *Pinellia ternata* (Thunb.) Makino., 15g  ·Dried body of *Gekko swinhonis* Guenther, 10g  ·Dried twigs of *Cinnamomum cassia* Presl, 10g  ·Dried peels of *Citrus reticulata* Blanco, 10g  ·Dried rhizomes of *Zingiber officinale* Rosc..12g | N | N |
| Gu N 2019 | Jianwei Yiai Powder | - | ·Dried herbs of *Solanum nigrum* L., 15g  ·Dried roots of *Sophora tonkinensis* Gagnep., 30g  ·Dried herbs of *Scutellaria barbata* D.Don, 30g  ·Dried herbs of *Hedyotis diffusa* Willd., 30g  ·Dried body of *Scolopendra subspinipes* mutilans L. Koch, 10g  ·Dried rhizomes of *Pinellia ternata* (Thunb.) Makino., 12g  ·Shell of *Arca subcrenata* Lischke, 30g  ·Dried roots of *Aucklandia lappa* Decne., 6g  ·Dried sclerotia of *Poria cocos* (Schw.) Wolf, 15g  ·Dried stems of *Spatholobus suberectus* Dunn, 30g  ·Dried roots of *Codonopsis pilosula* (Franch.) Nannf., 15g  ·Dried rhizomes of *Atractylodes macrocephala* Koidz., 15g  ·Dried roots and rhizomes of *Glycyrrhiza uralensis* Fisch..6g | N | N |
| Guo R 2014 | Wenyang Sanjie Decoction | Prepared by Department of Pharmacy, The First Affiliated Hospital of Zhengzhou University | ·Dried roots of *Codonopsis pilosula* (Franch.) Nannf., 20g  ·Dried roots of *Astragalus mongholicus* Bunge., 20g  ·Dried rhizomes of *Atractylodes macrocephala* Koidz., 20g  ·Dried sclerotia of P*oria cocos* (Schw.) Wolf, 15g  ·Dried fruits of *Ligustrum lucidum* Ait., 15g  ·Dried rhizomes of *Pinellia ternata* (Thunb.) Makino., 12g  ·Dried herbs of *Hedyotis diffusa* Willd., 15g  ·Dried pseudobulb of *Cremastra appendiculata* (D.Don) Makino, 15g  ·Dried peels of *Citrus reticulata* Blanco,10g  ·Dried roots of *Actinidia chinensis* Planch. var. hispida C.F.Liang, 20g  ·Membrane of *Chickens Gizzard*, 15g  ·Dried roots and rhizomes of *Glycyrrhiza uralensis* Fisch..10g | N | N |
| Hu FS 2012 | Yiqi Huoxue Jiedu Formula | - | ·Dried roots of *Astragalus mongholicus* Bunge., 30g  ·Dried roots of *Codonopsis pilosula* (Franch.) Nannf., 15g  ·Dried roots of *Pseudostellaria heterophylla* (Miq.) Pax, 30g  ·Dried rhizomes of *Atractylodes macrocephala* Koidz., 10g  ·Dried sclerotia of *Poria cocos* (Schw.) Wolf, 10g  ·Dried fruits of *Lycium barbarum* L., 10g  ·Dried fruits of *Ligustrum lucidum* Ait., 15g  ·Dried seeds of *Cuscuta chinensis* Lam., 15g  ·Dried stems of *Spatholobus suberectus* Dunn, 30g  ·Dried roots of *Paeonia lactiflora* Pall., 10g  ·Dried rhizomes of *Curcuma phaeocaulis* Val., 10g  ·Dried rhizomes of *Paris polyphylla* Smith var.yunnanensis (Franch.) Hand.-Mazz., 15g  ·Dried herbs of *Hedyotis diffusa* Willd., 30g  ·Dried roots of *Actinidia chinensis* Planch. var. hispida C.F.Liang, 15g | N | N |
| Huang J 2017 | Jianpi Yangwei Formula | - | ·Dried herbs of *Hedyotis chrysotricha* (Palib.) Merr., 30g  ·Dried rhizomes of *Smilax china* L., 30g  ·Seed of *Coix lacryma-jobi* L., 20g  ·Dried rhizomes of *Dioscorea opposita* Thunb., 15g ·Dried roots of *Codonopsis pilosula* (Franch.) Nannf., 10g  ·Dried rhizomes of *Atractylodes macrocephala* Koidz., 10g  ·Dried sclerotia of *Poria cocos* (Schw.) Wolf, 10g  ·Dried roots of *Aucklandia lappa* Decne., 10g  ·Dried roots of *Angelica sinensis* (Oliv.) Diels, 10g  ·Dried roots of *Paeonia lactiflora* Pall., 10g  ·Dried roots and rhizomes of *Glycyrrhiza uralensis* Fisch., 3g | N | N |
| Huang JQ 2014 | Jianpi Huayu Decoction | - | ·Dried roots of *Codonopsis pilosula* (Franch.) Nannf., 15g  ·Dried rhizomes of *Atractylodes macrocephala* Koidz., 10g  ·Dried herbs of *Taraxacum mongolicum* Hand.-Mazz., 15g  ·Dried stems of *Perilla frutescens* (L.) Britt., 10g  ·Dried roots and rhizomes of *Nardostachys jatamansi* DC., 10g  ·Dried seeds of *Trichosanthes kirilowii* Maxim., 10g  ·Dried rhizomes of *Pinellia ternata* (Thunb.) Makino., 10g  ·Dried body of *Gekko swinhonis* Guenther, 5g  ·Dried rhizomes of *Curcuma phaeocaulis* Val., 10g  ·Dried fruits of *Crataegus pinnatifida* Bge., 10g  ·Dried roots and rhizomes of *Rheum officinale* Baill., 3g  ·Dried barks of *Magnolia officinalis* Rehder & E.H.Wilson, 10g  ·Membrane of Chickens Gizzard., 5g | N | N |
| Jiang F 2021 | Jiedu Sanjie Formula | - | ·Dried herbs of *Taraxacum mongolicum* Hand.-Mazz., 15g  ·Dried ears of *Prunella vulgaris* L., 15g  ·Dried flowers of *Lonicera japonica* Thunb., 10g  ·Dried fruits of *Forsythia suspensa* (Thunb.) Vahl, 10g  ·Dried roots of *Angelica sinensis* (Oliv.) Diels, 8g  ·Dried roots of *Scrophularia ningpoensis* Hemsl., 8g  ·Dried roots of *Isatis indigotica* Fort., 6g  ·Dried body of *Stiff silkworm*, 6g  ·Resin of *Commiphora myrrha* Engl., 5g  ·Dried body of *Buthus martensii* Karsch, 3g  ·Dried thorns of *Gleditsia sinensis* Lam..3g | N | N |
| Jiao JQ 2019 | Modified Xuezheng Decoction | Prepared by Department of TCM Pharmacy, Shangluo Central Hospital. | ·Dried rhizomes of *Sparganium stoloniferum* Buch.-Ham., 15g  ·Dried rhizomes of *Curcuma phaeocaulis* Val.,15g  ·Dried herbs of *Hedyotis diffusa* Willd., 15g  ·Dried rhizomes of *Smilax china* L., 15g  ·Dried roots of *Angelica sinensis* (Oliv.) Diels, 10g  ·Dried roots of *Paeonia lactiflora* Pall., 10g  ·Resin of *Boswellia carterii* Birdw., 5g  ·Resin of *Commiphora myrrha* Engl., 5g  ·Dried twigs of *Cinnamomum cassia* Presl.5g | N | N |
| Li DC 2016 | Fuzheng Kangai Formula | - | ·Dried rhizomes of *Atractylodes macrocephala* Koidz.,15g  ·Dried roots of *Morinda officinalis* How, 20g  ·Dried fruits of *Lycium barbarum* L., 30g  ·Dried rhizomes of *Drynaria fortunei* (Kunze) J.Sm.,20g  ·Prepared roots of *Rehmannia glutinosa* Libosch., 15g  ·Dried leaf of *Epimedium brevicornu* Maxim., 30g  ·Dried fruits of *Cornus officinalis* Sieb. et Zucc., 30g  ·Dried roots and rhizomes of *Panax ginseng* C. A. Mey., 50g  ·Dried bark of *Eucommia ulmoides* Oliv., 30g  ·Dried fruits of *Psoralea corylifolia* L., 20g  ·Dried bark of *Cinnamomum cassia* Presl, 5g  ·Dried roots of *Angelica sinensis* (Oliv.) Diels, 10g  ·Dried rhizomes of *Curculigo orchioides* Gaertn., 10g | N | N |
| Li DH 2020 | Shunqi Yiwei Decoction | - | ·Dried roots of *Bupleurum chinense* DC.,  ·Dried fruits of *Citrus aurantium* L.,  ·Dried roots of *Paeonia lactiflora* Pall.,  ·Dried roots and rhizomes of *Glycyrrhiza uralensis* Fisch.,  ·Dried sclerotia of *Poria cocos* (Schw.) Wolf,  ·Dried rhizomes of *Atractylodes macrocephala* Koidz.,  ·Dried roots of *Codonopsis pilosula* (Franch.) Nannf.. | N | N |
| Liu LF 2015 | Jianpi Xiaozheng Formula | - | ·Dried roots of *Astragalus mongholicus* Bunge., 30g  ·Dried rhizomes of *Smilax china* L., 30g  ·Dried herbs of *Hedyotis chrysotricha* (Palib.) Merr., 30g  ·Seed of *Coix lacryma-jobi* L., 20g  ·Dried roots of *Codonopsis pilosula* (Franch.) Nannf., 20g  ·Dried sclerotia of *Poria cocos* (Schw.) Wolf, 20g  ·Dried rhizomes of *Dioscorea opposita* Thunb., 20g  ·Dried rhizomes of *Sparganium stoloniferum* Buch.-Ham., 15g  ·Dried rhizomes of *Curcuma phaeocaulis* Val.,15g  ·Dried rhizomes of *Atractylodes macrocephala* Koidz., 10g  ·Dried roots of *Aucklandia lappa* Decne., 10g  ·Dried peels of *Citrus reticulata* Blanco, 10g  ·Dried roots of *Angelica sinensis* (Oliv.) Diels, 10g  ·Dried roots of *Paeonia lactiflora* Pall..10g | N | N |
| Liu ZW 2019 | Jianpi Huayu Formula | - | ·Membrane of Chickens Gizzard,5  ·Dried roots of *Codonopsis pilosula* (Franch.) Nannf.,15  ·Dried barks of *Magnolia officinalis* Rehder & E.H.Wilson, 10  ·Dried rhizomes of *Atractylodes macrocephala* Koidz., 10  ·Dried roots and rhizomes of *Rheum officinale* Baill., 3  ·Dried herbs of *Taraxacum mongolicum* Hand.-Mazz., 15  ·Dried fruits of *Crataegus pinnatifida* Bge., 15  ·Dried stems of *Perilla frutescens* (L.) Britt., 10  ·Dried rhizomes of *Curcuma phaeocaulis* Val., 10  ·Dried roots and rhizomes of *Nardostachys jatamansi* DC., 10  ·Dried body of *Gekko swinhonis* Guenther,5  ·Dried seeds of *Trichosanthes kirilowii* Maxim., 15  ·Dried rhizomes of *Pinellia ternata* (Thunb.) Makino..10 | N | N |
| Long YJ 2021 | Yiqi Fuyuan Formula | Prepared by Department of Paste Prescription, Hunan Province Directly Affiliated TCM Hospital. | ·Dried roots of *Astragalus mongholicus* Bunge., 300g  ·Dried roots of *Codonopsis pilosula* (Franch.) Nannf.,300g  ·Dried rhizomes of *Atractylodes macrocephala* Koidz., 200g  ·Dried roots of *Aucklandia lappa* Decne., 90g  ·Dried peels of *Citrus reticulata* Blanco, 90g  ·Dried sclerotia of *Poria cocos* (Schw.) Wolf, 200g  ·Dried roots of *Angelica sinensis* (Oliv.) Diels, 150g  ·Dried seeds of *Ziziphus jujuba* Mill. var. Spinosa (Bunge) Hu ex H. F. Chou, 200g  ·Dried rhizomes of *Dioscorea opposita* Thunb., 200g  ·Fresh rhizomes of *Zingiber officinale* Rosc.,90 g  ·Dried seeds of *Nelumbo nucifera* Gaertn., 120g  ·Dried roots and rhizomes of *Glycyrrhiza uralensis* Fisch..90g | N | N |
| Qin XG 2012 | Modified Buzhong Yiqi Decoction | - | ·Dried roots of *Astragalus mongholicus* Bunge., 30g  ·Dried roots of *Codonopsis pilosula* (Franch.) Nannf., 20g  ·Dried rhizomes of *Atractylodes macrocephala* Koidz., 10g  ·Dried roots and rhizomes of *Glycyrrhiza uralensis* Fisch., 15g  ·Dried roots of *Angelica sinensis* (Oliv.) Diels, 10g  ·Dried rhizomes of *Ligusticum chuanxiong* Hort., 10g  ·Dried peels of *Citrus reticulata* Blanco, 6g  ·Dried rhizomes of *Curcuma phaeocaulis* Val., 6g  ·Dried herbs of *Hedyotis diffusa* Willd., 30g  ·Seed of *Coix lacryma-jobi* L..20g | N | N |
| Sun B 2020 | Fuzheng Kangai Formula | - | ·Dried roots of *Codonopsis pilosula* (Franch.) Nannf., 20g  ·Dried rhizomes of *Atractylodes macrocephala* Koidz., 12g  ·Dried roots of *Bupleurum chinense* DC., 12g  ·Dried roots of *Paeonia lactiflora* Pall., 10g  ·Dried sclerotia of *Poria cocos* (Schw.) Wolf, 12g  ·Dried peels of *Citrus reticulata* Blanco, 12g  ·Dried rhizomes of *Pinellia ternata* (Thunb.) Makino., 10g  ·Dried rhizomes of *Ligusticum chuanxiong* Hort., 10g  ·Dried fruits of *Citrus aurantium* L., 12g  ·Dried fruits of *Ligustrum lucidum* Ait., 15g  ·Dried body of *Ganoderma lucidum* (Leyss.ex Fr.) Karst., 15g  ·Dried sprouts of *Hordeum vulgare* L., 15g  ·Dried sprouts of *Setaria italica* (L.) Beauv., 15g  ·Dried rhizomes of *Paris polyphylla* Smith var.yunnanensis (Franch.) Hand.-Mazz., 15g  ·Dried herbs of *Scutellaria barbata* D.Don, 15g  ·Dried herbs of *Hedyotis diffusa* Willd., 15g  ·Dried roots and rhizomes of *Glycyrrhiza uralensis* Fisch..6g | N | N |
| Wang Y 2018 | Guishao Liujunzi Decoction | Prepared by Department of TCM Pharmacy, Jiangsu Cancer Hospital, and Department of Pharmacy, Affiliated Hospital of Nanjing University of Chinese Medicine. | ·Dried roots of *Angelica sinensis* (Oliv.) Diels, 10g  ·Dried roots of *Paeonia lactiflora* Pall., 10g  ·Dried roots of *Codonopsis pilosula* (Franch.) Nannf., 15g  ·Dried sclerotia of *Poria cocos* (Schw.) Wolf, 15g  ·Dried rhizomes of *Atractylodes macrocephala* Koidz., 10g  ·Dried rhizomes of *Dioscorea opposita* Thunb., 15g  ·Seed of *Coix lacryma-jobi* L., 10g  ·Dried peels of *Citrus reticulata* Blanco, 6g  ·Dried rhizomes of *Pinellia ternata* (Thunb.) Makino., 10g  ·Dried herbs of *Hedyotis diffusa* Willd., 20g  ·Dried herbs of *Scutellaria barbata* D.Don, 15g  ·Dried rhizomes of *Sparganium stoloniferum* Buch.-Ham., 10g  ·Dried rhizomes of *Curcuma phaeocaulis* Val., 10g  ·Dried roots and rhizomes of *Glycyrrhiza uralensis* Fisch., 3g | N | N |
| Xie WS 2019 | Wendan Decoction | Supplied by Tianjiang pharmaceutical Co. Ltd., and prepared by Department of TCM Pharmacy, Shanxi Traditional Chinese Medicine Hospital. | ·Dried rhizomes of *Pinellia ternata* (Thunb.) Makino., 9g  ·Dried shavings of *Bambusa tuldoides* Munro, 12g  ·Dried fruits of *Citrus aurantium* L.,15g  ·Dried peels of *Citrus reticulata* Blanco, 10g  ·Dried sclerotia of *Poria cocos* (Schw.) Wolf, 15g  ·Dried roots and rhizomes of *Glycyrrhiza uralensis* Fisch., 3g  ·Fresh rhizomes of *Zingiber officinale* Rosc., 3g  ·Dried fruits of *Ziziphus jujuba* Mill., 3g | N | N |
| Yang Y 2018 | Shenyu Yangwei Decoction | - | ·Dried roots and rhizomes of *Panax ginseng* C. A. Mey.,30g  ·Dried fruits of *Cornus officinalis* Sieb. et Zucc.,10g  ·Dried stems of *Dendrobium nobile* Lindl.,15g  ·Dried roots and rhizomes of *Salvia miltiorrhiza* Bge., 20g | N | N |
| Yu D 2019 | Modified Shengyang Yiwei Decoction | - | ·Dried roots of *Astragalus mongholicus* Bunge., 30g  ·Dried roots of *Codonopsis pilosula* (Franch.) Nannf., 30g  ·Dried rhizomes of *Atractylodes macrocephala* Koidz., 15g  ·Dried sclerotia of *Poria cocos* (Schw.) Wolf, 20g  ·Dried rhizomes of *Alisma orientale* (Sam.) Juzep., 10g  ·Dried roots of *Paeonia lactiflora* Pall., 15g  ·Dried roots of *Angelica sinensis* (Oliv.) Diels, 15g  ·Dried herbs of *Scutellaria barbata* D.Don, 15g  ·Dried herbs of *Hedyotis diffusa* Willd.,15g  ·Dried rhizomes of *Curcuma phaeocaulis* Val., 10g  ·Dried seeds of *Prunus persica* (L.) Batsch, 10g  ·Dried rhizomes of *Pinellia ternata* (Thunb.) Makino., 10g  ·Dried peels of *Citrus reticulata* Blanco, 10g  ·Dried roots and rhizomes of *Glycyrrhiza uralensis* Fisch., 5g | N | N |
| Yuan KM 2011 | Modified Shengyang Yiwei Decoction | Prepared by Department of Pharmacy, Wuxi Hospital of Traditional Chinese medicine | ·Dried roots of *Codonopsis pilosula* (Franch.) Nannf., 10g  ·Dried rhizomes of *Atractylodes macrocephala* Koidz., 10g  ·Dried sclerotia of *Poria cocos* (Schw.) Wolf, 10g  ·Seed of *Coix lacryma-jobi* L., 10g  ·Dried rhizomes of *Pinellia ternata* (Thunb.) Makino., 10g  ·Dried peels of *Citrus reticulata* Blanco, 5g  ·Dried sclerotia of *Polyporus umbellatus* (Pers.) Fries,10g  ·Dried rhizomes of *Dioscorea opposita* Thunb., 10g  ·Dried sprouts of *Setaria italica* (L.) Beauv. , 15g  ·Dried sprouts of *Hordeum vulgare* L. , 15g  ·Dried sclerotia of *Poria cocos* (Schw.) Wolf with hostwood, 10g  ·Dried leaf of *Eriobotrya japonica* (Thunb.) Lindl., 10g  ·Dried roots and rhizomes of *Glycyrrhiza uralensis* Fisch., 6g | N | N |
| Yuan M 2018 | Yiqi Jiedu Formula | Prepared by Department of Pharmacy, Henan Province Hospital of TCM. | ·Dried roots and rhizomes of *Panax ginseng* C. A. Mey., 10g  ·Dried body of *Gekko swinhonis* Guenther, 20g  ·Dried rhizomes of *Paris polyphylla* Smith var.yunnanensis (Franch.) Hand.-Mazz., 10g  ·Dried rhizomes of *Pinellia ternata* (Thunb.) Makino., 12g  ·Dried peels of *Citrus reticulata* Blanco, 12g  ·Dried barks of *Magnolia officinalis* Rehder & E.H.Wilson, 12g  ·Dried roots and rhizomes of *Glycyrrhiza uralensis* Fisch..6g | N | N |
| Zhai YR 2019 | Huayu Jiedu Formula | Prepared by Orient Pharmaceutical Co.,Ltd, Henan Academy of Chinese Medicine | ·Dried roots and rhizomes of *Panax ginseng* C. A. Mey.,  ·Dried body of *Gekko swinhonis* Guenther,  ·Dried rhizomes of *Paris polyphylla* Smith var.yunnanensis (Franch.) Hand.-Mazz.,  ·Dried rhizomes of *Pinellia ternata* (Thunb.) Makino.,  ·Dried peels of *Citrus reticulata* Blanco,  ·Dried barks of *Magnolia officinalis* Rehder & E.H.Wilson,  ·Dried roots and rhizomes of *Glycyrrhiza uralensis* Fisch., | N | N |
| Zhang H 2021 | Modified Shiquan Dabu Decoction | - | ·Dried roots of *Astragalus mongholicus* Bunge., 30g  ·Seed of *Coix lacryma-jobi* L., 15g  ·Dried rhizomes of *Atractylodes macrocephala* Koidz., 10g  ·Dried sclerotia of *Poria cocos* (Schw.) Wolf, 10g  ·Dried roots of *Angelica sinensis* (Oliv.) Diels, 10g  ·Dried rhizomes of *Pinellia ternata* (Thunb.) Makino., 10g  ·Dried roots of *Paeonia lactiflora* Pall., 10g  ·Prepared roots of *Rehmannia glutinosa* Libosch., 10g  ·Dried rhizomes of *Sparganium stoloniferum* Buch.-Ham., 10g  ·Dried rhizomes of *Curcuma phaeocaulis* Val., 10g  ·Dried rhizomes of *Ligusticum chuanxiong* Hort., 10g  ·Dried roots and rhizomes of *Panax ginseng* C. A. Mey., 6g  ·Dried bark of *Cinnamomum cassia* Presl, 3g  ·Fruits of *Amomum villosum* Lour., 3g  ·Dried roots and rhizomes of *Glycyrrhiza uralensis* Fisch..3g | N | N |
| Zhang HO 2021 | Modified Chaihu Shugan Powder;  Modified Liujunzi Decoction;  Modified Yiwei Decoction;  Huangqi Zhuyu Daotan Decoction;  Modified Fuzi Lizhong Decoction;  Modified Shiquan Dabu Decoction | - | Modified Chaihu Shugan Powder:  ·Dried roots of *Bupleurum chinense* DC., 10g  ·Dried rhizomes of *Cyperus rotundus* L., 10g  ·Dried fruits of *Citrus aurantium* L., 10g  ·Dried rhizomes of *Atractylodes macrocephala* Koidz., 10g  ·Dried roots of *Paeonia lactiflora* Pall., 10g  ·Dried rhizomes of *Ligusticum chuanxiong* Hort., 10g  ·Dried rhizomes of *Pinellia ternata* (Thunb.) Makino., 10g  ·Dried seeds of *Oroxylum indicum* (L.) Vent., 6g  ·Dried fruits of *Gardenia jasminoides* Ellis, 6g  ·Fruits of *Amomum villosum* Lour., 4.5g,  ·Dried roots and rhizomes of *Glycyrrhiza uralensis* Fisch., 3g;  Modified Liujunzi Decoction:  ·Dried roots of *Codonopsis pilosula* (Franch.) Nannf., 15g,  ·Seed of *Coix lacryma-jobi* L., 15g,  ·Dried sclerotia of *Poria cocos* (Schw.) Wolf, 10g,  ·Dried rhizomes of *Atractylodes macrocephala* Koidz., 10g,  ·Dried fruits of *Citrus aurantium* L., 10g,  ·Dried rhizomes of *Pinellia ternata* (Thunb.) Makino., 10g,  ·Dried rhizomes of *Kaempferia galanga* L., 10g,  ·Dried peels of *Citrus reticulata* Blanco, 6g,  ·Fruits of *Amomum villosum* Lour., 6g,  ·Dried roots and rhizomes of *Glycyrrhiza uralensis* Fisch., 3g；  Modified Yiwei Decoction:  ·Dried roots of *Adenophora stricta* Miq., 15g  ·Dried rhizomes of *Dioscorea opposita* Thunb., 15g  ·Fresh roots of *Rehmannia glutinosa* Libosch., 15g  ·Dried roots of *Ophiopogon japonicus* (L.f) Ker-Gawl., 10g  ·Dried rhizomes of *Polygonatum odoratum* (Mill.) Druce, 10g  ·Dried roots of *Paeonia lactiflora* Pall., 10g  ·Dried stems of *Dendrobium nobile* Lindl., 10g  ·Dried barks of *Paeonia suffruticosa* Andr., 10g  ·Dried rhizomes of *Anemarrhena asphodeloides* Bge., 6g  ·Dried leaf of *Nelumbo nucifera* Gaertn., 6g  ·Dried roots and rhizomes of *Glycyrrhiza uralensis* Fisch., 3g;  Huangqi Zhuyu Daotan Decoction:  ·Dried faeces of *Trogopterus xanthipes* Milne-Edwards, 10g  ·Dried rhizomes of *Ligusticum chuanxiong* Hort., 10g  ·Dried rhizomes of *Sparganium stoloniferum* Buch.-Ham., 10g  ·Dried rhizomes of *Curcuma phaeocaulis* Val., 10g  ·Dried rhizomes of *Corydalis yanhusuo* W.T.Wang, 10g  ·Dried roots of *Paeonia lactiflora* Pall., 10g  ·Dried fruits of *Citrus aurantium* L., 10g  ·Dried rhizomes of *Pinellia ternata* (Thunb.) Makino., 10g  ·Dried rhizomes of *Cyperus rotundus* L., 10g  ·Dried seeds of *Prunus persica* (L.) Batsch, 6g ·Dried rhizomes of *Arisaema erubescens* (Wall.) Schott, 6g  ·Seed of *Coix lacryma-jobi* L., 20g  ·Dried roots and rhizomes of *Glycyrrhiza uralensis* Fisch., 3g;  Modified Fuzi Lizhong Decoction:  ·Dried roots of *Codonopsis pilosula* (Franch.) Nannf., 15g  ·Dried roots of *Angelica sinensis* (Oliv.) Diels, 15g  ·Dried sclerotia of *Poria cocos* (Schw.) Wolf, 10g  ·Dried rhizomes of *Atractylodes macrocephala* Koidz., 10g  ·Dried rhizomes of *Curcuma phaeocaulis* Val., 10g  ·Dried roots of *Angelica sinensis* (Oliv.) Diels, 10g  ·Dried rhizomes of *Pinellia ternata* (Thunb.) Makino., 10g  ·Dried roots of *Paeonia lactiflora* Pall., 10g  ·Prepared subroots of *Aconitum carmichaelii* Debx., 6g  ·Dried rhizomes of *Zingiber officinale* Rosc., 6g  ·Dried roots and rhizomes of *Glycyrrhiza uralensis* Fisch., 3g;  Modified Shiquan Dabu Decoction:  ·Dried roots of *Astragalus mongholicus* Bunge. 15g,  ·Dried roots and rhizomes of *Panax ginseng* C. A. Mey. 6g,  ·Dried rhizomes of *Atractylodes macrocephala* Koidz. 10g,  ·Dried sclerotia of *Poria cocos* (Schw.) Wolf 10g,  ·Dried roots of *Paeonia lactiflora* Pall.10g,  ·Dried roots of *Angelica sinensis* (Oliv.) Diels 10g,  ·Dried fruits of *Psoralea corylifolia* L. 10g,  ·Prepared roots of *Rehmannia glutinosa* Libosch. 10g,  ·Dried rhizomes of *Pinellia ternata* (Thunb.) Makino. 10g,  ·Dried rhizomes of *Sparganium stoloniferum* Buch.-Ham. 10g,  ·Dried rhizomes of *Curcuma phaeocaulis* Val. 10g,  ·Dried bark of *Cinnamomum cassia* Presl 3g,  ·Fruits of *Amomum villosum* Lour. 3g,  ·Dried roots and rhizomes of *Glycyrrhiza uralensis* Fisch. 3g. | N | N |
| Zhang HW 2021 | Shugan Yangwei Decoction | Prepared by Department of TCM Pharmacy, Gansu Provincial Cancer Hospital. | ·Dried roots of *Bupleurum chinense* DC., 10g  ·Dried roots of *Paeonia lactiflora* Pall., 15g  ·Dried roots of *Scutellaria baicalensis* Georgi, 10g  ·Dried fruits of *Citrus aurantium* L. Immaturus, 10g  ·Dried rhizomes of *Coptis chinensis* Franch., 6g  ·Dried rhizomes of *Zingiber officinale* Rosc., 6g  ·Dried rhizomes of *Pinellia ternata* (Thunb.) Makino., 10g  ·Dried roots of *Aucklandia lappa* Decne., 10g  ·Dried roots and rhizomes of *Rheum officinale* Baill., 6g  ·Dried roots and rhizomes of *Salvia miltiorrhiza* Bge., 20g  ·Dried fruits of *Melia toosendan* Sieb.et Zucc., 20g  ·Dried rhizomes of *Corydalis yanhusuo* W.T.Wang, 10g  ·Dried roots of *Codonopsis pilosula* (Franch.) Nannf., 10g  ·Dried seeds of *Alpinia katsumadai* Hayata, 10g  ·Dried roots and rhizomes of *Glycyrrhiza uralensis* Fisch., 6g | N | N |
| Zhang LH 2020 | Shengyang Yiwei Decoction | Supplied by Sanjiu Medical & Pharmaceutical Co., Ltd. | ·Dried roots of *Astragalus mongholicus* Bunge.,30g  ·Dried rhizomes of *Pinellia ternata* (Thunb.) Makino., 12g  ·Dried roots and rhizomes of *Panax ginseng* C. A. Mey.,15g  ·Dried roots and rhizomes of *Glycyrrhiza uralensis* Fisch., 9g  ·Dried roots of *Angelica pubescens* Maxim.f. biserrata Shan et Yuan, 10g  ·Dried roots of *Saposhnikovia divaricata* (Turcz.) Schischk., 10g  ·Dried roots of *Paeonia lactiflora*Pall., 10g  ·Dried roots and rhizomes of *Notopterygium incisum* Ting ex H.T.Chang, 10g  ·Dried peels of *Citrus reticulata* Blanco, 6g  ·Dried sclerotia of *Poria cocos* (Schw.) Wolf, 10g  ·Dried roots of *Bupleurum chinense* DC., 6g  ·Dried rhizomes of *Alisma orientale*（Sam.）Juzep., 10g  ·Dried rhizomes of *Atractylodes macrocephala* Koidz., 12g  ·Dried rhizomes of *Coptis chinensis* Franch., 3g | N | N |
| Zhang ZP 2019 | Erteng Sanjie Capsule | Prepared by Department of TCM Pharmacy, Shanxi Traditional Chinese Medicine Hospital. | ·Dried roots of *Pseudostellaria heterophylla* (Miq.) Pax,  ·Dried rhizomes of *Atractylodes macrocephala* Koidz.,  ·Seed of *Coix lacryma-jobi* L.,  ·Dried rhizomes of *Pinellia ternata* (Thunb.) Makino.,  ·Dried peels of *Citrus reticulata* Blanco,  ·Fruits of *Amomum villosum* Lour.,  ·Dried body of *Gekko swinhonis* Guenther,  ·Dried viens of *Sargentodoxa cuneata* (Oliv.) Rehd. et Wils.,  ·Dried rhizomes of *Smilax china* L.,  ·Dried viens of *Vitis heyneana* Roem. et Schult,  ·Dried roots of *Actinidia chinensis* Planch. var. hispida C.F.Liang,  ·Dried ears of *Prunella vulgaris* L.,  ·Shell of *Ostrea gigas* Thunberg,  ·Dried rhizomes of *Curcuma phaeocaulis* Val.,  ·Dried seeds of *Areca catechu* L.,  ·Dried roots and rhizomes of *Glycyrrhiza uralensis* Fisch., | N | N |
| Zhao H 2011 | Kun Shen Granule | Prepared by Department of Pharmacy, Shandong University of Traditional Chinese Medicine. | ·Dried body of *Laminariajaponica* Aresch.,  ·Dried roots of *Actinidia chinensis* Planch. var. hispida C.F.Liang,  ·Dried herbs of *Agrimonia pilosa* Ledeb.,  ·Dried roots and rhizomes of *Panax ginseng* C. A. Mey.. | N | N |
| Zhao XN 2016 | Jianpi Huayu Formula | - | ·Dried roots of *Pseudostellaria heterophylla* (Miq.) Pax, 15g  ·Dried rhizomes of *Atractylodes macrocephala* Koidz., 15g  ·Dried rhizomes of *Paris polyphylla* Smith var.yunnanensis (Franch.) Hand.-Mazz., 15g  ·Dried roots and rhizomes of *Salvia miltiorrhiza* Bge., 15g  ·Dried herbs of *Scutellaria barbata* D.Don, 15g  ·Dried herbs of *Salvia chinensis* Benth., 15g  ·Dried herbs of *Hedyotis diffusa* Willd., 10g  ·Dried sclerotia of *Poria cocos* (Schw.) Wolf, 10g  ·Dried herbs of *Solanum nigrum* L., 10g  ·Dried stems of *Dendrobium nobile* Lindl., 10g  ·Dried rhizomes of *Dioscorea opposita* Thunb..20g | N | N |
| Zhao YY 2021 | Wenyang Jianpi Decoction | - | ·Dried roots of *Pseudostellaria heterophylla* (Miq.) Pax, 20g  ·Dried herbs of *Hedyotis diffusa* Willd., 15g  ·Dried rhizomes of *Atractylodes macrocephala* Koidz., 15g  ·Dried rhizomes of *Curcuma phaeocaulis* Val., 15g  ·Dried herbs of *Solanum nigrum* L., 15g  ·Dried sprouts of *Setaria italica* (L.) Beauv. , 15g  ·Dried sprouts of *Hordeum vulgare* L. ,15g  ·Dried rhizomes of *Pinellia ternata* (Thunb.) Makino., 10g  ·Dried sclerotia of *Poria cocos* (Schw.) Wolf, 10g  ·Dried peels of *Citrus reticulata* Blanco, 10g  ·Dried fruits of *Citrus aurantium* L., 10g  ·Dried twigs of *Cinnamomum cassia* Presl, 10g  ·Dried rhizomes of *Zingiber officinale* Rosc., 6g  ·Dried roots and rhizomes of *Glycyrrhiza uralensis* Fisch..4g | N | N |
| Zhong XS 2021 | Jianpi Fuzheng Xiaoliu Formula | - | ·Dried roots of *Pseudostellaria heterophylla* (Miq.) Pax, 20g  ·Seed of *Coix lacryma-jobi* L., 20g  ·Dried rhizomes of *Atractylodes macrocephala* Koidz.,15g  ·Dried herbs of *Hedyotis diffusa* Willd., 15g  ·Dried rhizomes of *Smilax china* L., 15g  ·Dried herbs of *Scutellaria barbata* D.Don, 15g  ·Dried rhizomes of *Sparganium stoloniferum* Buch.-Ham., 15g  ·Dried rhizomes of *Curcuma phaeocaulis* Val., 15g  ·Dried sclerotia of *Poria cocos* (Schw.) Wolf,12g  ·Dried rhizomes of *Dioscorea opposita* Thunb., 10g  ·Dried roots of *Angelica sinensis* (Oliv.) Diels, 10g  ·Dried fruits of *Crataegus pinnatifida* Bge., 10g  ·Dried roots and rhizomes of *Glycyrrhiza uralensis* Fisch., 6g  ·Membrane of Chickens Gizzard.6g | N | N |
| Zhong ZJ 2019 | Modified Shenling Baizhu Decoction | - | ·Dried peels of *Citrus reticulata* Blanco, 8g  ·Dried roots of *Bupleurum chinense* DC., 10g  ·Dried roots of *Platycodon grandiflorus* (Jacq.) A.DC., 10g  ·Dried roots and rhizomes of *Glycyrrhiza uralensis* Fisch.,10g  ·Dried seeds of *Nelumbo nucifera* Gaertn., 10g  ·Fruits of *Amomum villosum* Lour., 10g  ·Dried roots of *Angelica sinensis* (Oliv.) Diels, 10g  ·Dried rhizomes of *Atractylodes macrocephala* Koidz., 15g  ·Dried seeds of *Dolichos lablab* L., 15g  ·Dried sclerotia of *Poria cocos* (Schw.) Wolf, 15g  ·Dried rhizomes of *Dioscorea opposita* Thunb., 18g  ·Dried roots of *Codonopsis pilosula* (Franch.) Nannf., 20g  ·Dried roots of *Astragalus mongholicus* Bunge., 30g  ·Seed of *Coix lacryma-jobi* L..30g | N | N |
| Zhu XY 2011 | Jianpi Yiqi Decoction | - | ·Dried roots of *Astragalus mongholicus* Bunge., 30g  ·Dried fruits of *Crataegus pinnatifida* Bge., 30g  ·Scorch-fried medicated leaven, 30g  ·Dried sprouts of *Hordeum vulgare* L. , 30g  ·Dried roots of *Glehnia littoralis* Fr. Schmidtex Miq., 15g  ·Dried roots of *Adenophora stricta* Miq., 15g  ·Dried rhizomes of *Polygonatum kingianum* Coll.et Hemsl., 15g  ·Dried peels of *Citrus reticulata* Blanco, 10g  ·Dried rhizomes of *Pinellia ternata* (Thunb.) Makino., 10g  ·Fruits of *Citrus medica* L. var. sarco- dactylis Swingle, 10g  ·Dried barks of *Magnolia officinalis* Rehder & E.H.Wilson, 10g  ·Membrane of Chickens Gizzard, 10g  ·Fruits of *Amomum villosum* Lour., 6g  ·Dried fruits of *Amomum kravanh* Pierre ex Gagnep., 6g  ·Dried roots and rhizomes of *Glycyrrhiza uralensis* Fisch., 3g | N | N |
